# AI and semantic ontology for personalized activity eCoaching in healthy lifestyle recommendations: a meta-heuristic approach

**DOI:** 10.1186/s12911-023-02364-4

**Published:** 2023-12-01

**Authors:** Ayan Chatterjee, Nibedita Pahari, Andreas Prinz, Michael Riegler

**Affiliations:** 1 Department of Information and Communication Technology, Centre for E-Health, University of Agder, Grimstad, Norway; 2 Department of Holistic Systems, Simula Metropolitan Center for Digital Engineering (SimulaMet), Oslo, Norway; 3 Department of Computer Science and Engineering, Maulana Abul Kalam Azad University of Technology, Kolkata, India

**Keywords:** eCoach, Physical activity, Autoregression, Time-series, Residual error minimization, Ensemble, Interval prediction, Ontology, Personalized recommendation

## Abstract

**Background:**

Automated coaches (eCoach) can help people lead a healthy lifestyle (e.g., reduction of sedentary bouts) with continuous health status monitoring and personalized recommendation generation with artificial intelligence (AI). Semantic ontology can play a crucial role in knowledge representation, data integration, and information retrieval.

**Methods:**

This study proposes a semantic ontology model to annotate the AI predictions, forecasting outcomes, and personal preferences to conceptualize a personalized recommendation generation model with a hybrid approach. This study considers a mixed activity projection method that takes individual activity insights from the univariate time-series prediction and ensemble multi-class classification approaches. We have introduced a way to improve the prediction result with a residual error minimization (REM) technique and make it meaningful in recommendation presentation with a Naïve-based interval prediction approach. We have integrated the activity prediction results in an ontology for semantic interpretation. A SPARQL query protocol and RDF Query Language (SPARQL) have generated personalized recommendations in an understandable format. Moreover, we have evaluated the performance of the time-series prediction and classification models against standard metrics on both imbalanced and balanced public PMData and private MOX2-5 activity datasets. We have used Adaptive Synthetic (ADASYN) to generate synthetic data from the minority classes to avoid bias. The activity datasets were collected from healthy adults (n = 16 for public datasets; n = 15 for private datasets). The standard ensemble algorithms have been used to investigate the possibility of classifying daily physical activity levels into the following activity classes: sedentary (0), low active (1), active (2), highly active (3), and rigorous active (4). The daily step count, low physical activity (LPA), medium physical activity (MPA), and vigorous physical activity (VPA) serve as input for the classification models. Subsequently, we re-verify the classifiers on the private MOX2-5 dataset. The performance of the ontology has been assessed with reasoning and SPARQL query execution time. Additionally, we have verified our ontology for effective recommendation generation.

**Results:**

We have tested several standard AI algorithms and selected the best-performing model with optimized configuration for our use case by empirical testing. We have found that the autoregression model with the REM method outperforms the autoregression model without the REM method for both datasets. Gradient Boost (GB) classifier outperforms other classifiers with a mean accuracy score of 98.00%, and 99.00% for imbalanced PMData and MOX2-5 datasets, respectively, and 98.30%, and 99.80% for balanced PMData and MOX2-5 datasets, respectively. Hermit reasoner performs better than other ontology reasoners under defined settings. Our proposed algorithm shows a direction to combine the AI prediction forecasting results in an ontology to generate personalized activity recommendations in eCoaching.

**Conclusion:**

The proposed method combining step-prediction, activity-level classification techniques, and personal preference information with semantic rules is an asset for generating personalized recommendations.

**Supplementary Information:**

The online version contains supplementary material available at 10.1186/s12911-023-02364-4.

## Key Contributions to the Literature

- This conceptual study has hypothesized a personalized hybrid activity recommendation generation method in an activity eCoach prototype system.
- The daily collection of real-time activity data with a medical-grade wearable activity sensor (e.g., MOX2-5) has served as an input for the activity eCoaching session. Recommendation generation aims to motivate participants to meet their personal activity goals and reduce sedentary time. The individual preference datasets (such as goal setting, response type, and interaction type) have been helpful for the meaningful delivery of personalized recommendation messages.
- The autoregression model with residual error minimization technique has shown the potential to improve forecasting performance in time series. Besides, the ensemble approach has been helpful for daily activity level classification on activity sensor data.
- We have introduced the application of the ADASYN sampling algorithm for data balancing to avoid prediction biases in machine learning classifiers. Moreover, we have used Mathew’s coefficient (MCC) metric to cross-verify prediction biases.
- Semantic ontology has been used to logically represent personal preference data, prediction and classification outcomes, knowledge reasoning, and querying. Combined with a defined ruleset, the SPARQL queries help to generate personalized physical activity recommendations.

## Introduction

This section encompasses the background, motivation, current state-of-the-art, and the study's objectives. Additionally, it includes a qualitative comparison with prior research to highlight the uniqueness and innovation brought by this study.

### Background

About 60% to 85% of people live a sedentary lifestyle worldwide [1]. The collective effects of the sedentary lifestyle are related to several adverse health outcomes, including increased risk of lifestyle diseases, such as obesity, diabetes type II, high blood pressure, depression, and cardiovascular threats [1–10]. Regular physical activity has a positive impact on preventing and managing lifestyle diseases. Compared with people with adequate exercise, people with inadequate activity have an increased risk of death by 20% to 30% [10]. An automatic health coach may help people to manage a healthy lifestyle with ubiquitous personalized health state monitoring (e.g., physical activity, nutrition, healthy habits) and tailored recommendations [11–14]. A coaching process can be “In-person” or “Technology-driven” (via Telematic means) [12]. In-person coaching with manual activity tracking and personalized recommendations is inefficient and repetitive. Therefore, in this regard, an automatic coach can be more efficient. An eCoach system tries to involve users proactively in an ongoing collaborative dialogue to support planning and encourage effective goal management using personalized health and wellness status monitoring and thereby, recommendation generation to meet the lifestyle goal [14].

Recommendation technology, a decision-making approach under complex information environments can be classified as rule-based and data-driven [15–17]. The data-driven recommendations use AI algorithms. In contrast, rule-based recommendation technology uses binary logic in a symbolic form to present knowledge in IF–THEN or IF-ELSEIF-THEN rules and infer new knowledge with the reasoning method. A knowledge base (KB) is maintained to store and access such rules and associated messages. Rules can be specified in the form of propositional logic, decision tree, relational algebra, and description logic. Rule-based systems are modular, intelligible, and easy to manage; however, they suffer from symbol grounding problems [16]. The data-driven approach experiences a lack of sufficient data, high computing power, lack of interpretability, re-training for new cases, personalization, and cold-start. Therefore, to overcome the failings of data-driven and rule-based recommendations, a hybrid approach can be useful.

Description logic is the formal knowledge representation of ontology language (e.g., Web Ontology Language (OWL), which balances clarity, complexity, and effectiveness of knowledge description and knowledge reasoning. Semantic Web Rule Language (SWRL), and SPARQL are well-accepted query languages in semantic ontology [4]. An ontology is a formal description of knowledge within a domain and its relationships following a hierarchical structure. Other methods of knowledge representation are thesaurus, topic maps, and logical models. Still, unlike relational database schemas, ontologies express relationships and allow users to join or link multiple concepts together with the following elements: individuals/objects, classes, attributes, relationships, and axioms [4].

### Motivation

Behavior and health are strongly connected. Reduction of a sedentary lifestyle with increased physical activity needs self-motivation, self-correlation, and self-management. Tudor-Locke et al. [18] and Matthews et al. [19] showed that human activity varies between the weekends and weekdays. Gardner et al. [20] acknowledged that self-monitoring, and reforming the social and physical environment are the most encouraging strategies for human behavior change besides recommending environmental reorganization, persuasion, and education to improve self-regulation skills. Intervention design to improve physical activity levels and reduce sedentary time varies significantly in content and effectiveness [20–22]. Mobile applications used to improve young people's physical activity should include personalized feedback and provide guidance [14]. Only a few available mHealth applications for physical activity have been evaluated, and the evidence is of inferior quality [14].

In the digital activity recommendation system, a tracker is maintained to record daily step count, metabolic equivalent of tasks, kilocalories, and distance to reduce sedentary behavior. Such digital recommendation systems consist of a data collection module, an AI module, and a recommendation generation or decision module. Data are captured over time and analyzed with AI algorithms to generate real-time feedback to accomplish personal activity goals. The decision module recommends changing a person's behavior, daily routine, and activity plan [20]. A walking tracker smartphone app can measure individual activity levels and enable self-monitoring [23, 24]. Most of the modern consumer-based activity sensors (e.g., Fitbit, Actigraph, MOX2-5, Pedometer, Garmin, and smartwatches (e.g., Apple, Samsung, Huawei)) based smartphone apps contain a variety of behavior change models or theories [25–28]; however, they experience lack of a genuine eCoaching flavor. A meta-analysis from Qiu et al. [29] and Stephenson et al. [30] concluded that using a pedometer has a small but significant effect on reducing sedentary time. Just wearing an activity tracker (even without any form of guidance) can stimulate the passion for performing physical activities to improve the quality of life.

Only a few studies have investigated the use of actionable, data-driven predictive models [31]. Dijkhuis et al. [32] analyzed Hanze University's personalized physical activity coaching with AI algorithms to improve sedentary lifestyles. They collected daily step data to train AI classifiers to estimate the probability of achieving hourly step goals and followed by feedback generation with a web-based coaching application. Hansel et al. [33] designed a fully automated web-based coaching program. They used pedometer-based activity or step monitoring in a random group of Type 2 diabetes and abdominal obesity patients to increase their physical activity. Pessemier et al. [34] used raw accelerometer data for individual activity recognition, accepted personal preferences for activity recommendation planning, and generated personalized recommendations with tag-based recommender and rule-based filter. Amorim et al. [35] and Oliveira et al. [36] performed activity monitoring with a Fitbit activity sensor on a group of random trials. They accomplished a statistical analysis to discover the efficacy of a multimodal physical activity intervention with supervised exercises, health coaching, and activity monitoring on physical activity levels of patients suffering from chronic, nonspecific low back pain. Petsani et al. [37] designed an eCoach system for older people to increase faithfulness to exergame-based physical activities. They followed the inclusion of eCoaching guidelines set by the human therapists/doctors or a familiar person chosen by the user who can access their persistent health and wellness data and involve in the coaching process. They remarked that health eCoaching is a complex process that needs careful planning and integration of different scientific domains, such as psychology, computer science, health informatics, and medical science. Braber et al. [38] incorporated the eCoaching concept in personalized diabetes management where lifestyle data (e.g., dietary intake, physical activity, glycemic value) were recorded and integrated with clinical rules to give customized coaching to improve adherence to lifestyle recommendations.

Chatterjee et al. [12] focused on creating a meaningful, context-specific ontology to model non-intuitive, raw, and unstructured observations of personal and person-generated health data (e.g., sensors, interviews, questionnaires) using semantic metadata to create a logical abstraction for rule-based health risk prediction and thereby, personalized lifestyle recommendation generation in a health eCoach system. Villalonga et al. [39] conceptualized an ontology-based automated reasoning model for generating personalized motivational messages for activity coaching considering behavioral characteristics. Thus, ontology can be a good alternative for rule-based decision-making with robust design flexibility in object-oriented design paradigms.

Improvement of physical activity in combination with wearable activity sensors and digital activity trackers, eCoach features can be promising and motivating to its participants. The application of AI to eCoaching is new. Therefore, real-time data analysis and, thereby, the generation of personalized recommendations with eCoaching is missing in existing literature with the following search string in well-reputed PubMed or Medline database: with a search string: *((ecoach OR e-coach) AND (activity monitoring) AND (Healthy lifestyle or lifestyle) AND (activity or physical activity or exercise) AND (Sensor or activity sensor or activity tracker) AND (recommendation or recommendation generation) AND (data driven or data-driven or classification or prediction or regression or forecasting or rule-based or rule based or ruleset or knowledge base or knowledge-based or hybrid))*. Different activity monitoring and lifestyle coaching smartphone applications are available online; however, they are too generic and lack appropriate design and development guidelines, and eCoaching features [12].

### State-of-the-art

The state-of-the-art is to generate personalized recommendations using AI and interpretable semantic rules to motivate participants to achieve their activity goals. A goal type can be of two types – short-term goals (e.g., weekly) and/or long-term goals (e.g., monthly). Success in short-term goals (STG) attainment may help in achieving long-term goals (LTG) when the LTGs are the summation of STGs.

Our assumed hypothesis is that an eCoach system can generate meaningful, automatic, and personalized recommendation plans to accomplish individual lifestyle goals. To prove the concept, we have conceptualized the design of the ActieCoach prototype system for physical activity as a study case. ActieCoach can collect activity and personal preference data from actual participants with wearable activity sensors, questionnaires, and self-reported forms, respectively, and thereby, process collected data to forecast daily step count, classify individual activity levels, and combine the outcomes in an ontology model for semantic knowledge representation to generate of personalized recommendations with a query engine against a defined semantic rule set. The semantic rules in an ontology can show a direction to enhance the understandability of recommendation generation with IF-ELSE conditions in a logical tree structure. Most activity trackers, involving mobile apps and smart wearable devices (e.g., smartwatches), predict future activity in terms of "steps" as a point prediction with time-series forecasting, probabilistic approaches, or specific rules. However, point prediction is a very abstract concept. Therefore, a probabilistic interval prediction approach may be encouraging. Preliminary research has been found on sensor data with AI technology and combining the predictive analysis result with semantic rules for hybrid recommendation generation. Moreover, this research adds arguments to attain ethical aspects of AI by addressing a collection of ethical data, data governance, testing for bias, explainable AI, and continuous model improvement with incremental model designing.

This study is novel as no similar work has been published as revealed from the literature search. Recommendation technology has a broad application domain. We have considered studies that are only related to lifestyle recommendations, either personal or group-level. A qualitative comparison between our study and the related studies has been made in Table 1 based on the following parameters: hybrid recommendations (data-driven and rule-based), ontology modeling, interval prediction, observation with activity sensors, preference settings, and logical recommendation generation. The high-level descriptions of the used terminologies are specified in Additional file 3: Appendix A.1. The study by Pessemier et al. [34] focused on recommendation generation at the “Community” level; however, our research targets activity coaching and recommendation generation at the “Personal” level.

**Table 1:** A qualitative comparison between our study and the related healthy lifestyle recommendation studies

Study	Hybrid recommendation?	Ontology modeling	Interval prediction	Real-time observation with an activity sensor	Preference settings	Logical recommendation generation
*Our work*	*Yes*	*Yes*	*Yes*	*Yes*	*Yes*	*Yes*
[12]	Rule-based	No	No	No	No	Yes
[29]	No	No	No	Yes	No	No
[30]	No	No	No	Yes	No	No
[32]	Data-driven	No	No	Yes	No	No
[33]	No	No	No	Yes	No	No
[34]	Yes	No	No	Yes	Yes	No
[35]	No	No	No	Yes	No	No
[36]	No	No	No	Yes	No	No
[37]	No	No	No	No	No	No
[38]	No	No	No	No	No	No
[39]	Rule-based	No	No	No	No	Yes

### Aim of the study

This theoretical and experimental evaluation study in laboratory settings addresses the following identified research questions associated with automatic and tailored recommendation generation for physical activity in our ActieCoach:

- *How to combine AI forecasting and classification outcomes in an ontology with semantics?*
- *Can residual error minimization technique improve the performance of time-series prediction?*
- *How to use AI technology with semantic rules in automatic activity coaching for personalized and understandable recommendation generation?*
- *How to handle data imbalances to avoid model biases?*

## Design of the eCoach system

**Fig. 1:**
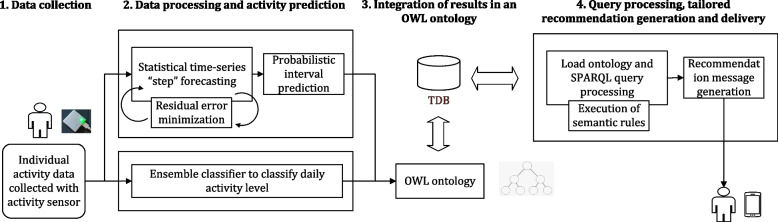
The proposed solution for ActieCoach

This section discusses the design of our ActieCoach prototype system. We followed an iterative and incremental approach to design and implement our eCoach prototype that follows a modular design pattern with four key modules (see Fig. 1): (a.) data collection, (b.) data processing and activity prediction, (c.) integrating the outcomes in an ontology and (d) recommendation generation based on SPARQL query processing. The data processing and activity prediction module is divided into two sub-modules: step forecasting and daily activity level classification (see Table 2). The individual data collection, step prediction, activity level classification, and personalized recommendation generation are continuous processes according to the eCoach feedback cycle. All the data and results were stored in an In-Memory tuple database (TDB) [5].

**Table 2:** The rules for “Activity Level” feature formation are based on standard guidelines for activity level classification [10]

Activity Level	Rule^a^	Score
Sedentary	((Steps < 5000) ∧ (VPA*2 + MPA) *7 < 90 ∧ LPA ≥ 0)) ˅ (Steps < 5000)	0
Low active	((Steps > 4999) ∧ (VPA*2 + MPA) *7 ≥ 90 ∧ (VPA*2 + MPA) *7 < 210) ˅ (Steps > 4999 ∧ Steps < 7500)	1
Active	((Steps > 4999) ∧ (VPA*2 + MPA) *7 ≥ 210 ∧ (VPA*2 + MPA) *7 < 300) ˅ (Steps > 7499 ∧ Steps < 10,000)	2
Medium active	((Steps > 4999) ∧ (VPA*2 + MPA) *7 ≥ 300 ∧ (VPA*2 + MPA) *7 < 360)) ˅ (Steps > 9999 ∧ Steps < 12,500)	3
Highly active	((Steps > 4999) ∧ (VPA*2 + MPA) *7 ≥ 360) ˅ (Steps > 12,499)	4

^a^MPA = 2VPA

We used a traditional wearable activity sensor for personal activity data collection following individual consent and ethical guidelines,, such as General Data Protection Regulation (GDPR) [40]. Participants were recruited on a voluntary basis. We prepared a set of questionnaires to collect personal preference data (e.g., activity goal setting, response type, and the nature interaction) for recommendation planning. Participants were allowed to view and update their preference information at any time. The classification sub-module classifies daily activity data into the following activity levels: sedentary, low physical activity (LPA), medium physical activity (MPA), and vigorous physical activity (VPA). The prediction sub-module is responsible for forecasting daily steps for the next 7-days based on the temporal pattern in individual step data. After semantic annotation with ontology, the respective module stores personal preference data and activity prediction and classification results in the TDB.

We designed a pipeline to automate the process with an incremental approach to handle real-time growing activity data with continuous machine learning model training, validation, and testing. The recommendation generation module executes a scheduler to query and process individual activity prediction results from the TDB database with a SPARQL query engine, on a regular basis. A KB has been maintained in the TDB. In the KB, all the semantic rules are stored for recommendation generation. Semantic rules consist of propositional variables using (IMPLIES), (NOT), (AND), and (OR) operations. The recommendation generation module triggers a logical rule of structure (A IMPLIES B). If some specific variables are inferred to be true, then some suggestions should be provided to the participants of the semantic data source. Following, individual recommendation data are updated in the ontology against a timestamp before storing it back in the database. The TDB database can be accessed periodically for personal preference data and the generation of individual recommendation messages or feedback data.

The design follows a modular microservice architecture. The exposed eCoach interfaces are protected with multi-factor authentication and authorization rules [41, 42]. The data processing and activity module is written in Python (V. 3.8.x) with the Flask Framework and Python libraries. The other modules are written in the Java (JDK 11 +) programming language with the SpringBoot Framework. Open-source Apache libraries (Jena, Jena Fuseki) [5] have been used for ontology implementation and eCoach service deployment.

### Ontology modeling and algorithm design for personalized recommendations

**Fig. 2:**
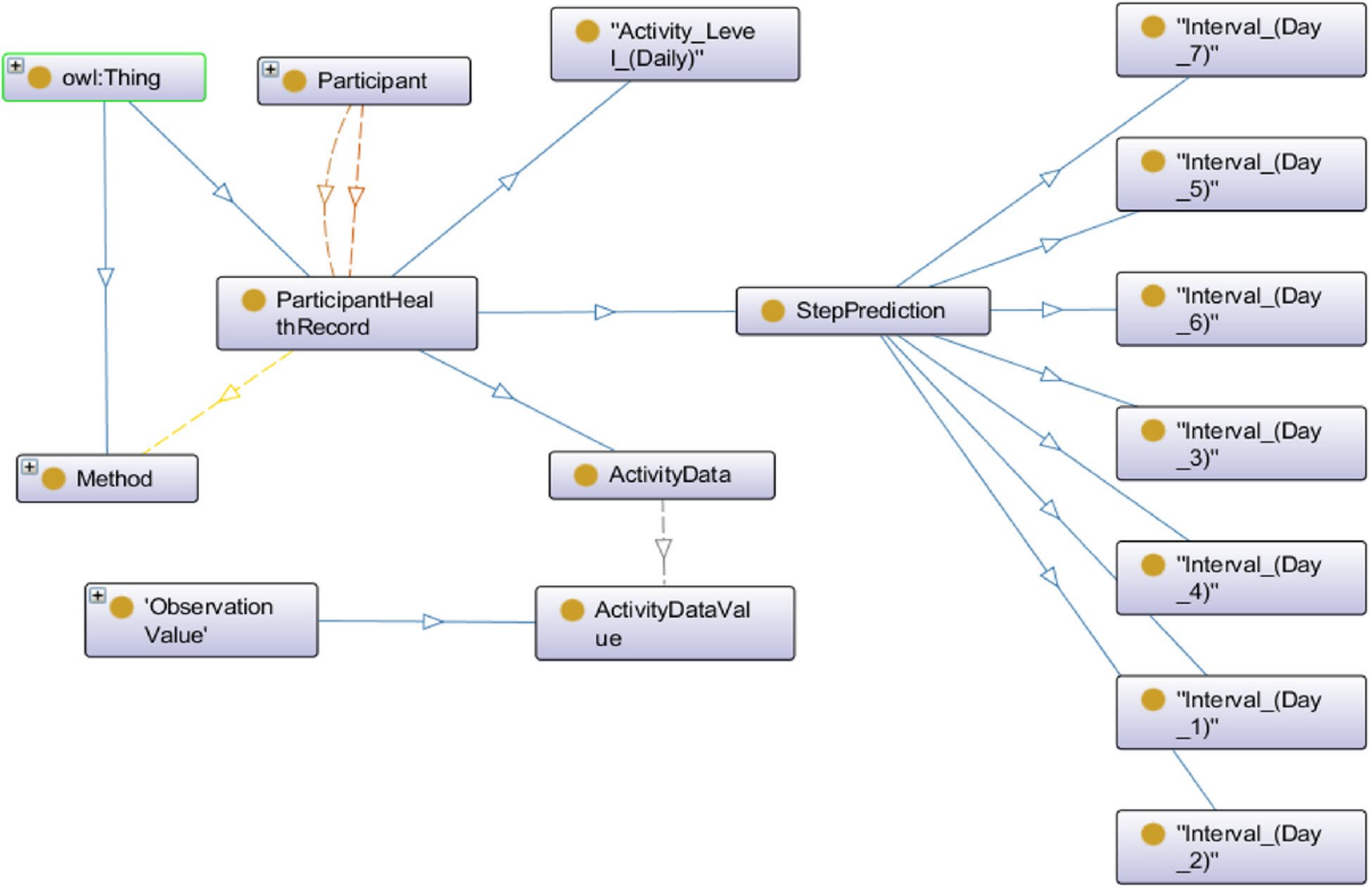
The high-level structure of the proposed ontology

**Fig. 3:**
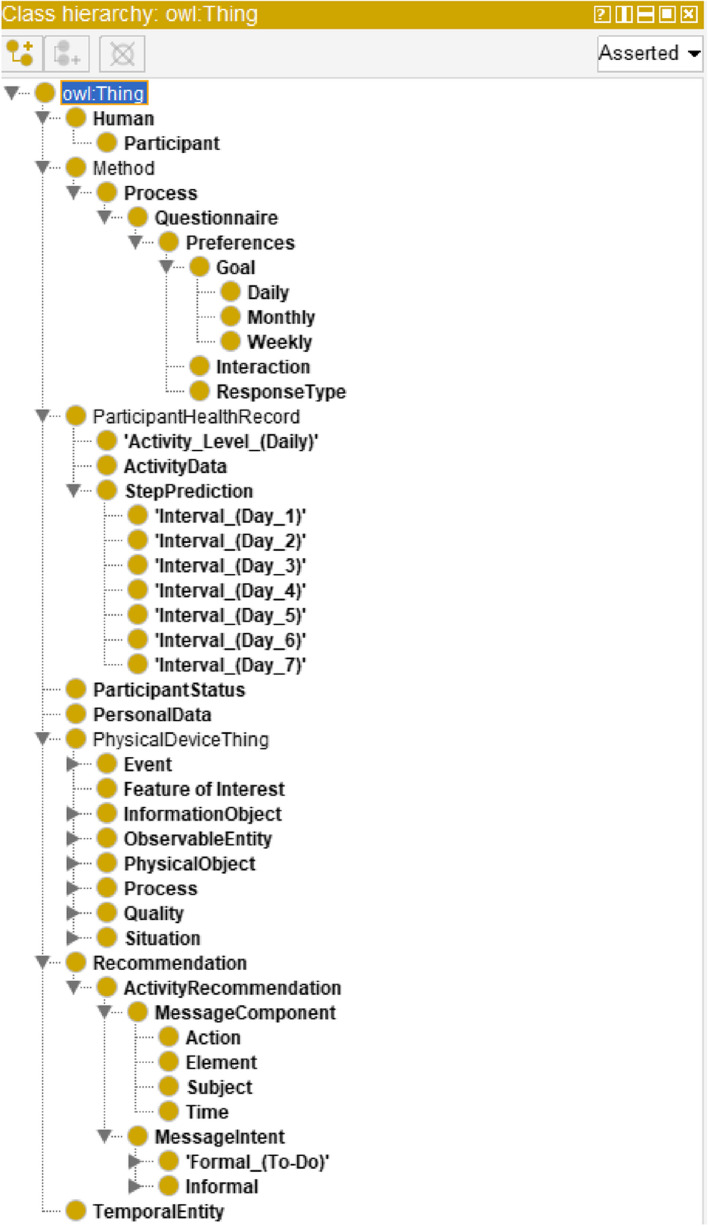
The asserted class hierarchy of our proposed ontology

The concept of ontology supports an open-world assumption knowledge representation style with the following elements: classes, individuals/objects, attributes or properties, relationships, and axioms [5, 43]. Properties are of two types: ObjectProperties and DataProperties. Each property has a domain range, restriction rule, restriction filter, and restriction type as Some (existential), Only (Universal), Min (Minimum Cardinality), Exact (Exact Cardinality), and Max (Max Cardinality) [43]. Owl: Thing acts as a super-class in an ontology class hierarchy [43]. The class diagram of a program written with object-oriented programming visually represents an ontology structure. An ontology follows a connected, acyclic, and directed tree structure [43]. Our ontology has been explained in Table 3 and its high-level structure is depicted in Fig. 2, using the OntoGraf tool plugged-in Protégé. The asserted class hierarchy of the ontology has been depicted in Fig. 3.

**Table 3:**
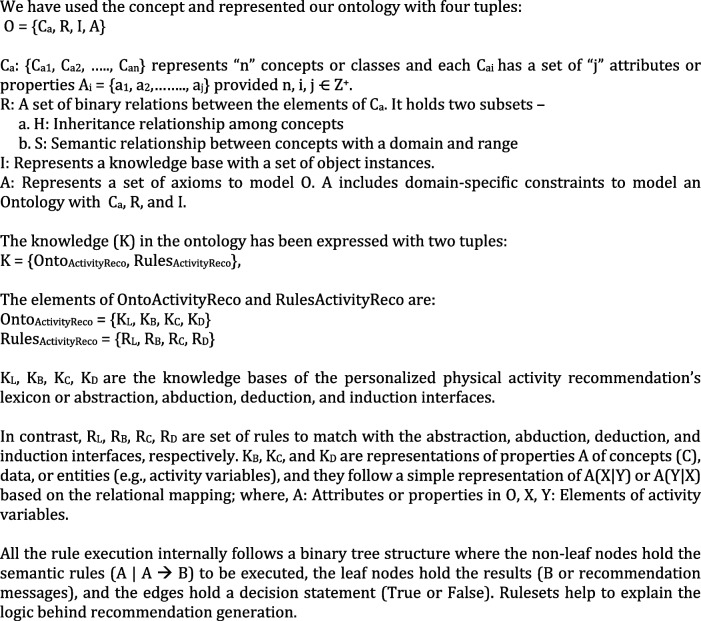
The Ontology structure and knowledge expression

The object properties, domain, range, property type, and cardinality of the ontology are defined in Table 4. The purpose of an ontology is the semantic representation of knowledge, reasoning, and rule-based decision-making with the generalization rules in the induction phase. The proposed ontology follows the following knowledge representation phases: abstraction or lexicon phase (L) for mapping rules, abduction phase (B) for hypothesis generation rule, deduction phase (C) for the operator-reduction rule, and induction phase (D) for generalization rule. The resultant recommendation generation tree (T) follows a binary structure, and the syntactic knowledge representation in T helps to address the understandability problem in personalized recommendation generation.

**Table 4:** Key object Properties, domain, range, and cardinality of the ontology

ObjectProperties	Domain	Range	Cardinality
hasHealthRecord	Participant	HealthRecord	Some
hasPersonalData	Participant	PersonalData	Some
hasPreferences	Participant	Preferences	Some
hasReceivedRecommendation	Participant	Recommendation	Some
hasStatus	Participant	ParticipantStatus	Some
hasbeenCollectedBy	ActivityData	ActivityDataValue	Some
hasTimeStamp	ActivityDataValue, Questionnaire, Recommendation, ParticipantHealthRecord	TemporalEntity	Some
has Measurement Capability	ActivityDevice	Measurement Capability	Only
hasOutput	ActivityDevice	Sensor Output	Some
observes	ActivityDevice	Property	Only
detects	ActivityDevice	Stimulus	Only
feature of interest	Observation	Feature of Interest	Only
observation result	Observation	Sensor Output	Only
observedBy	Observation	Sensor	Only
is property of	Property	Feature of Interest	Some
hasProperty	Feature of Interest	Property	Some
hasIntervalDay	Participant	StepPrediction	Some
hasActivityLevel	Participant	Activity_Level_(Daily)	Some

A set of propositional variables, logics, constants, and operators (such as NOT, AND, OR, IMPLIES, EQUIV, and quantifiers) are linked with Ontology representation and processing. The “EQUIV” refers to equivalence in logic or mathematics. In the context of a knowledge representation language such as OWL, it might be an equivalence relation, indicating that two entities have the same meaning or denotation. In this study, the recommendation generation aims to maximize weekly individual physical activity time to minimize sedentary time. The maximization problem to stay medium activate for a week (∑ days (1…0.7)) has been expressed in Table 5.

**Table 5:**
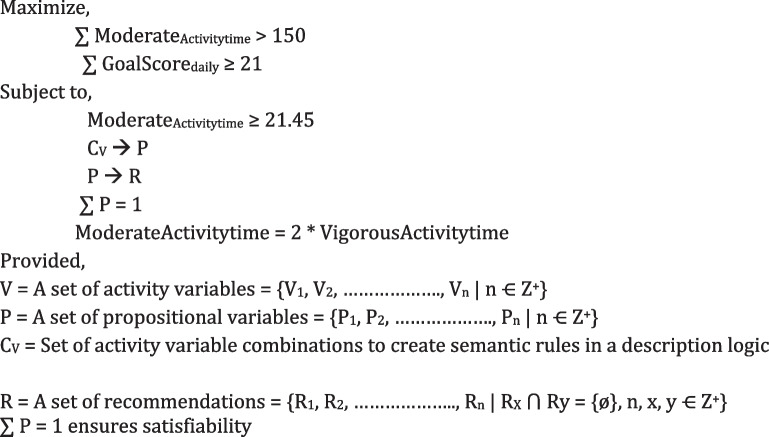
Expression for the activity maximization problem

According to the World Health Organization (WHO) guidelines, adults (age group:18–64) should do at least 150–300 min (2.5 – 5 h) of MPA; or at least 75–150 min of VPA or perform an equivalent combination of moderate and high-intensity activities within a week to stay active [10]. To determine the weekly score of personal goal achievement, we summed up the daily activity score (see Table 2). eCoaching aims at goal score maximization with constant activity monitoring and recommendation generation. To conceptualize the personalized recommendation generation in our eCoach system, we considered an example of personal preferences table (see Table 6). We integrated the designed and developed ontology model into the ActieCoach for the logical representation of forecasting, classification, and personal preferences results. Preferences can be following three types.

- Activity goal setting (e.g., kind of goals, direct vs. motivational goals, and generic vs. personalized goals).
- Response type (e.g., way to communicate extended health state, health state prediction, and tailored recommendations for activity coaching).
- The kind of interaction with ActieCoach (e.g., mode, frequency, and medium).

**Table 6:** Personalized preferences of individual participants for recommendation generation

Personal preference names	Selection of example preference values
Goal setting	Weekly score
Nature of goal	System defined—Generic [set by the WHO]
Frequency of recommendation delivery	Weekly
Target goal	To stay medium active for the entire week
Target score	21
Mode of recommendation	Text (e.g., push notification on the eCoach app.)
Time of recommendation	8:00 am

The generic activity goals are the activity guidelines set by the WHO.

Moreover, we have shown a direction to use the ontology for automatic rule-based personalized activity recommendation generation with SPARQL queries. The ontology has demonstrated an approach to annotating recommendation messages beyond the static verbatim form to describe its characteristics, metadata, and content information. The recommendation messages can be of two types: Formal and Informal (“To-Do”). Additionally, the rule base has helped to interpret the logic behind recommendation generation with logical (AND), (OR), and (NOT) operations. Additional file 3: Appendix A.2 describes a set of defined recommendation messages for ontology verification against the used datasets. Against each condition described in Additional file 3: Appendix A.3, the recommendation generation module will execute a SPARQL query to verify the type of recommended message to be delivered to individuals daily. This study divides eight semantic rules into activity level classification (7) and satisfiability (1).

Semantic rules are created to describe the relationships and limitations between different concepts or entities within our eCoach knowledge representation prototype system. These rules facilitate the capture of the intended meaning and the intended behavior of data, respectively, and enable deduction and inference capabilities. Creating semantic rules is complicated and requires an understanding of the domain, the participants, and the desired meaning. Other methods, including collaboration with domain experts and utilizing existing ontologies or knowledge bases, have also been beneficial in the creation of rules.

Time-stamped measurable parameters related to the activities of specific participants are obtained using SPARQL queries at preference-based intervals. The rules (1–7) in Additional file 3: Appendix A.3 assign truth values to variables to ensure consistency. We strengthened with the ontology reasoner that the correct recommendation message will be triggered for specific situations. However, it is essential to ensure that no variable pattern makes the entire rule unsatisfiable. We managed that only one message would be activated at a time. Here, we have a formal assurance that two "once a day" messages can neither be activated concurrently nor can there be a model output by the reasoner every time for every possible variable combination. Suppose we put the different variables used in the first seven rules in Additional file 3: Appendix A.3 into the propositional variables (see Additional file 3: Appendix A.2). In that case, we will have an exponential number of "possible participants". Since two messages cannot be triggered concurrently to meet the exact requirements, we have added a rule (Rule-8), and the variable used in the proposal starts "once a day". If (Rule-8) is false, the entire ruleset (considered as significant conjunction) will be set to false, and then there will be no model as output, and we will be able to "debug" our rules if needed. If it is set to true, we will have a formal assurance that no matter the true value we put in the rule base, two "once a day" messages will not be triggered simultaneously. All the rule execution internally follows a binary tree structure where the non-leaf nodes hold the semantic rules (A | A ➔ B) to be executed, the leaf nodes hold the results (B or recommendation messages), and the edges hold a decision statement (True or False). In this way, satisfiability, and understandability problems have been addressed in customized, evidence-based, and goal-oriented recommendation generations in ActieCoach. The proposed algorithm for the hybrid recommendation generation is described as follows:

#### Algorithm 1

A personalized hybrid activity recommendation generation method.
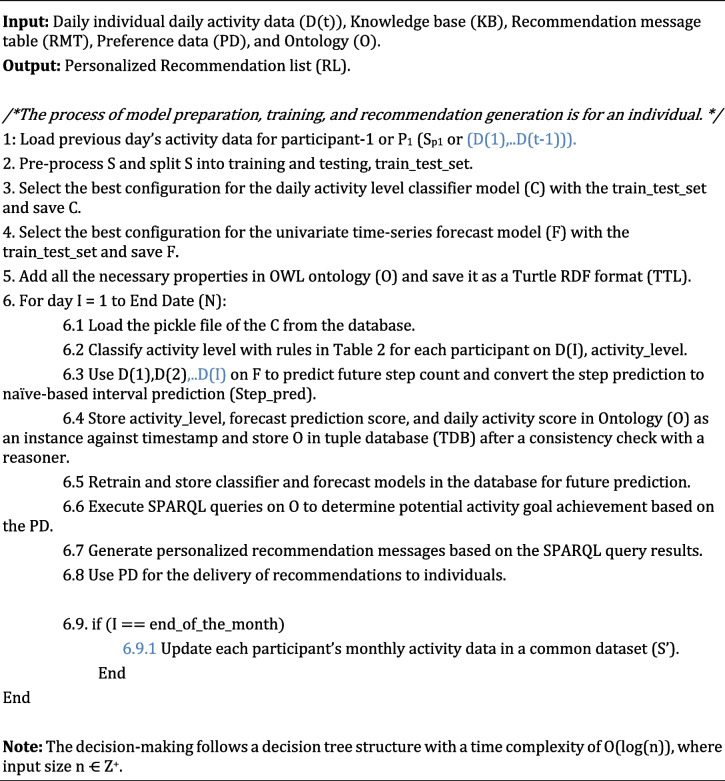


## Datasets

We used statistical forecasting and ensemble classification algorithms to analyze activity datasets for adults (age group: 18–64). Activity data for the elderly, children, athletes, bodybuilders, and pregnant women are beyond the scope. Our used datasets are imbalanced.

### PMData public datasets

We used the anonymous public PMData sports logging dataset for n = 15 adults (male: 12; female: 3) model training and testing participants. The activity dataset has been collected with Fitbit Versa 2 fitness smartwatch in the PMSys sports logging smartphone application. A detailed description of the dataset is provided by Vajira et al. [44]. We received records between 114–152 days per participant with a total volume of 2244 records. The dataset reveals multiple features related to physical activities, such as step count, sleep time, resting heart rate, type of exercise, restlessness, sedentary minutes, LPA, MPA, and VPA. We selected the “steps” meta-data file and excluded sleep-related features as sleep monitoring is not in the scope. We excluded Participant P-12 from the analysis due to missing LPA information.

### MOX2-5 private datasets

We collected anonymous activity data (539 records) from n = 16 adults (male: 12; female: 4) in Norway for 30–45 days using the MOX2-5 wearable activity sensor (CE certified) [45], following ethical guidelines and signed consent. Based on the Norwegian Centre for Research Data (NSD) approval, we collected and assessed personal data in this project following the data protection legislation. The attributes of the MOX2-5 data are in Additional file 3: Appendix A.3. The participant’s characteristics are recorded in Additional file 3: Appendix A.4. Informed or signed consent has been taken from all the participants. We have not disclosed any identifiable data of the participants using text, numbers, or figures. The relevant features obtained from the MOX2-5 sensors are—timestamp, activity intensity (IMA), sedentary seconds, weight-bearing seconds, standing seconds, LPA seconds, MPA seconds, VPA seconds, and steps per minute. The “step” and “IMA” are the most valuable and robust features of the MOX2-5 sensor-based datasets, as other attributes (except the timestamp) are almost derived (e.g., LPA, MPA, and VPA are derived from IMA as defined in Table 7). IMA has a strong relation with steps where steps are primarily involved as a measure for activities. In the MOX2-5 sensor, sedentary time refers to the non-activity duration, including leisure and sleep. The relation between sedentary and active (LPA/MPA/VPA) can be written as:

**Table 7:** Relation between IMA and activity level classification

Activity type	IMA range to determine physical activity types
LPA	0 ≤ IMA ≤ 400
MPA	401 ≤ IMA ≤ 800
VPA	IMA ≥ 801

1 $$\sum(\mathrm{sedentary},\;\mathrm{active},\;\mathrm{weight}-\mathrm{bearing},\;\mathrm{standing})=60\;\mathrm{seconds}$$

## Methods

This section describes an adopted methodology for feature selection, data labeling for classification, data processing with prediction and regression models, and model evaluation. We have followed the Standards for Reporting Implementation (StaRI) for this study (see Additional file 2: Appendix B). All methods have been carried out following the regulations and relevant guidelines in the “Ethical approval and consent to participate” section under Declarations.

### Feature selection

For the feature selection [46–50], we adopted well-established feature selection and feature ranking methods, such as SelectKBest, Recursive Feature Elimination (RFE), Principal Component Analysis (PCA), ExtraTreesClassifier, and correlation analysis. SelectKBest is a univariate feature selection and feature ranking method with statistical testing (e.g., chi-squared) [46–50]. RFE selects optimal features and assigns a rank after removing redundant features recursively [46–50]. PCA is an unsupervised data reduction method that uses linear algebra to reduce data dimensions. It ranks features based on variance ratio [46–50]. ExtraTreesClassifier is a bagging-based feature importance (or ranking) method [46–50]. Moreover, correlation analysis is a statistical method used to measure the strength of the linear relationship between two variables and compute their association [4]. A high correlation signifies a strong relationship between the two variables, and a low correlation means that the variables are weakly related [4]. The sample correlation coefficient (r) measures the closeness of association of the variables. "r" ranges from -1 to + 1, where -1 indicates a perfectly linear negative, i.e., inverse, correlation (sloping downward) and + 1 shows a linear positive correlation [4]. "r" close to 0 suggests little, if any, correlation. Correlation methods are of the following two types [4]: (a.) Pearson correlation: it evaluates the linear relationship between two continuous variables, (b.) Spearman correlation: It considers the monotonic or non-gaussian relationship. Our used datasets have shown a non-gaussian relationship with normality testing methods. The correlation analysis [4] with the “spearman” method revealed the strength of the linear relationship between features and helped to determine which feature to retain or not. We considered removing features if they showed a powerful dependency score (r >  = 0.72). The Shapiro–Wilk normality test [4] revealed that both data samples did not look like “Gaussian”. The normality test involved multiple univariate tests following the hypothesis testing method with *P-*value > α = 0.05 (i.e., the sample looks like Gaussian) and *P*-value < α = 0.05 (i.e., the sample does not look like Gaussian) [4]. “α” signifies the significance level. We have set a rule to eliminate participants' data that are less than one month redundant, noisy, incomplete, or missing. For time-series forecasting, we have considered univariate daily step counts from both datasets. Moreover, we have used the forward and backward filling methods to handle missing data.

### Combining features from datasets

**Fig. 4:**
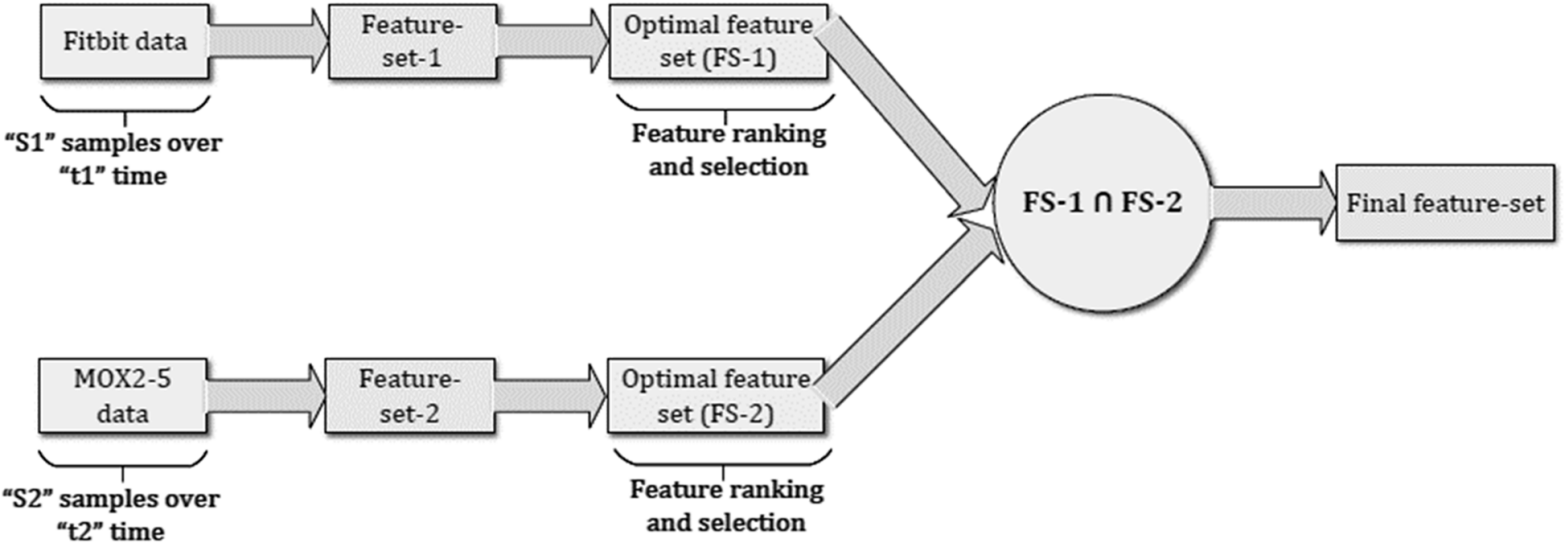
Combining features from both datasets to prepare the final feature set

First, we performed feature ranking and feature selection from public Fitbit datasets based on the adopted correlation method and created an optimal feature set (FS-1). Second, we have performed the same feature selection method on private MOX2-5 datasets and created an optimal feature set (FS-2). Then, we performed an intersection of FS-1 and FS-2 to create a common feature space (or final feature set) to make the transfer learning approaches relevant to this study (see Fig. 4).

### Data labeling for classification

**Fig. 5:**
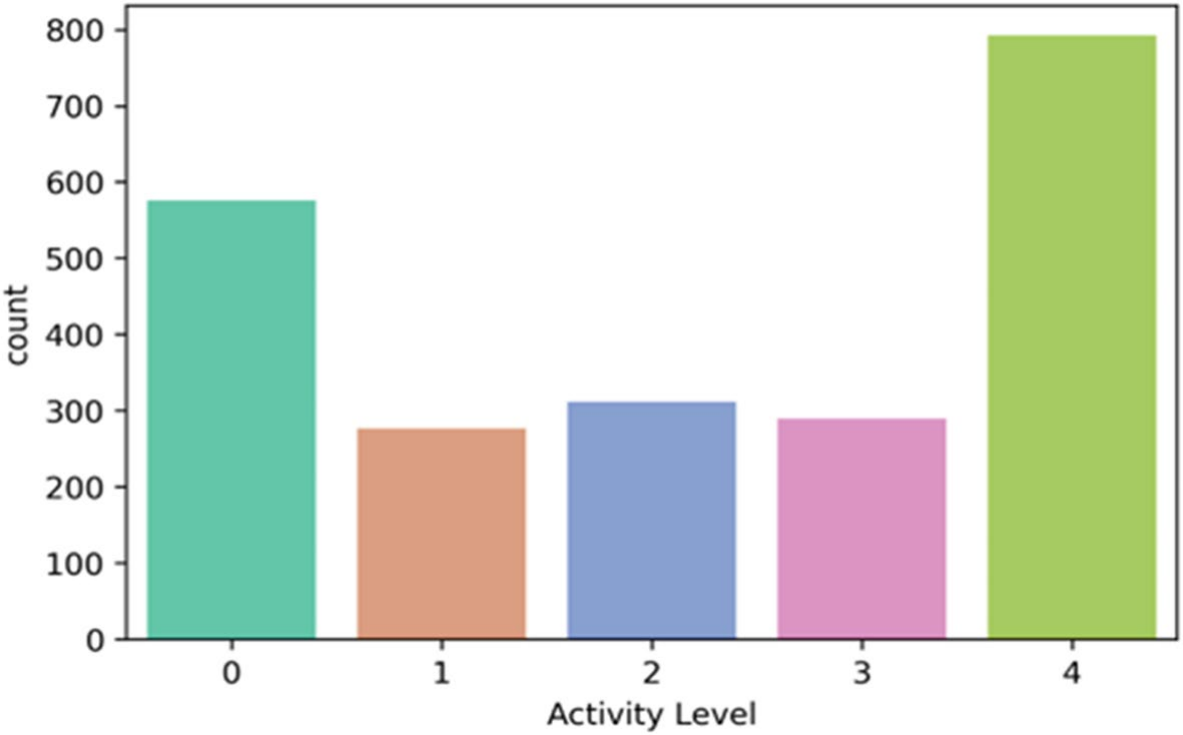
Class distribution for the public PMData datasets

**Fig. 6:**
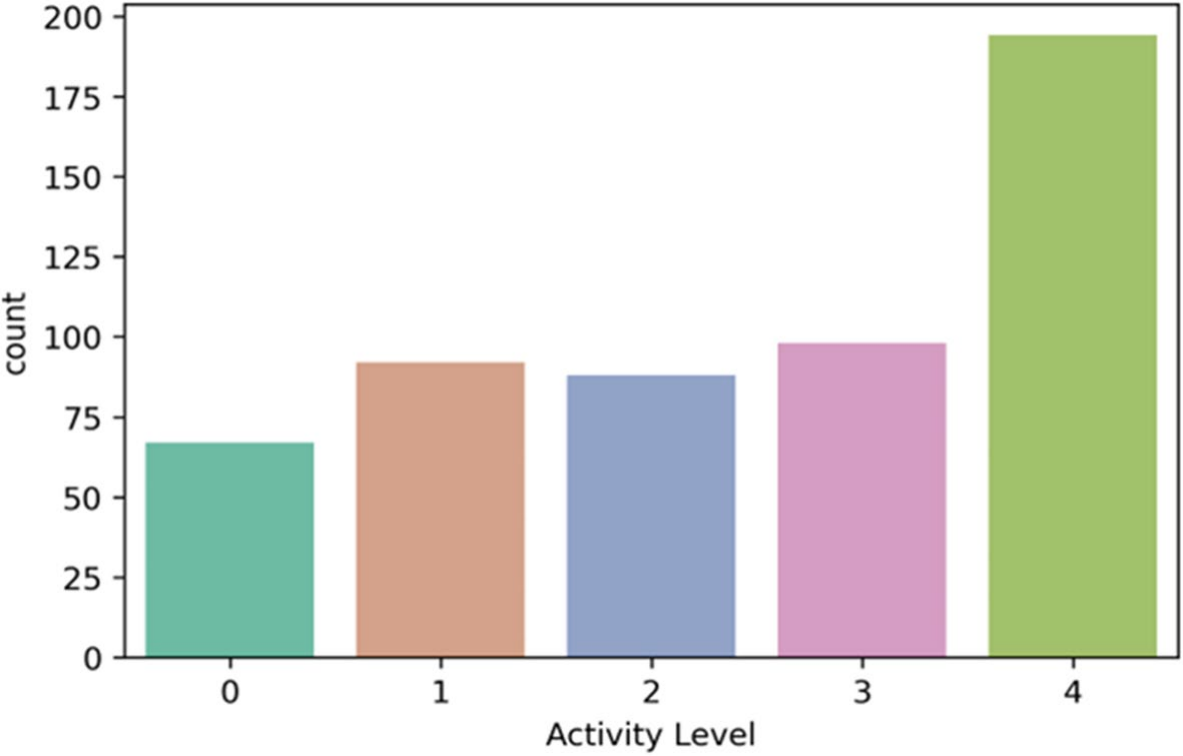
Class distribution for the private MOX2-5 datasets

The “Activity Level” feature represents five classes – sedentary (0), low active (1), active (2), medium active (3), and highly active (4). The rule for “Activity Level” feature class creation is defined in Table 2. In the multi-feature-based classification problem, we derived the feature class “active” based on the sedentary, LPA, MPA, and VPA following activity references for adults [10, 51, 52]. Features, such as age, gender, and weight are not in the scope of this study. The class distributions in multi-class classification for both datasets are depicted in Figs. 5 and 6.

### Data balancing for classification

Data balancing [53, 54] is crucial to addressing class imbalance and making sure that machine learning models are impartial, legitimate, and powerful. It increases the performance of the model, averts bias, enhances generalizability, facilitates better learning of features, prevents overfitting, and increases the model's stability to change in concept.

Synthetic Minority Over-sampling Technique (SMOTE) [55] is a well-known and widely used method for generating synthetic samples to remove class imbalance. It does this by creating virtual instances in the space of features of the minority class that are synthetic in nature, they are interpolated between the existing samples of the minority class. It chooses a random sample of minority classes and finds the k closest classes to it. It then produces synthetic samples that follow the line segments that connect the smaller sample and its neighbors. SMOTE can increase the percentage of the minority class and decrease the class disparity. ADASYN [56] is a supplement to SMOTE that addresses the deficiency of SMOTE in dealing with data that have a more intricate distribution of classes.

ADASYN provides an adaptive method that considers the local density distribution of the minority class. This method is intended to better represent the distribution of underlying data and provide a more effective method of addressing class imbalance. In this study, we have used the ADASYN method for data balancing for predictive analysis.

Let “X” be the feature matrix of the original dataset with n samples and m features, and y be the corresponding class labels. Let “X_minority” and “X_majority” represent the feature matrices of the minority and majority classes, respectively. The adopted steps for the ADASYN method have been summarized as follows:

- 1. Calculate the imbalance ratio (IR) as the ratio of the number of majority samples to the number of minority samples and the corresponding formula is:
  $$\mathrm{IR}=(\mathrm{number}\;\mathrm{of}\;\mathrm{majority}\;\mathrm{samples})/(\mathrm{number}\;\mathrm{of}\;\mathrm{minority}\;\mathrm{samples}).$$
- 2. Calculate the number of synthetic samples to generate for each minority sample based on the IR and the corresponding formula is:
  $$\mathrm N\_\mathrm{synthetic}=\mathrm{IR}\ast(\mathrm{number}\;\mathrm{of}\;\mathrm{minority}\;\mathrm{samples})$$
- 3. For each minority sample x_i_ in X_minority, calculate the Euclidean distance (dist) between xi and its k nearest neighbors in the feature space. The value of k is a user-defined parameter.
- 4. Calculate the relative contribution (rc) of each minority sample based on the normalized distance and the corresponding formula is:
  $${\mathrm{rc}(\mathrm{x}}_{\mathrm{i}})={\mathrm{dist}(\mathrm{x}}_{\mathrm{i}}) / \sum {\mathrm{dist}(\mathrm{x}}_{\mathrm{i}})$$
- 5. Calculate the density distribution (dd) for each minority sample and the corresponding formula is:
  $$\mathrm{dd}\left(\mathrm{xi}\right)=\mathrm{rc}\left({\mathrm{x}}_{\mathrm{i}}\right)/\sum \mathrm{rc}\left({\mathrm{x}}_{\mathrm{i}}\right)$$
- 6. Calculate the number of synthetic samples to generate for each minority sample based on the density distribution and the corresponding formula is:
  $$\mathrm{N}\_\mathrm{synthetic}\_\mathrm{i}=\mathrm{dd}({\mathrm{x}}_{\mathrm{i}})*\mathrm{N}\_\mathrm{synthetic}.$$
- 7. For each minority sample x_i_, generate N_synthetic_i synthetic samples by randomly interpolating between x_i_ and its k nearest neighbors.
- 8. Combine the original dataset X with the synthetic samples to form the augmented dataset X_augmented.
- 9. Apply a machine learning algorithm to the augmented dataset X_augmented for classification.

### Auto regression model with residual error minimization

Autoregression [57] is a time-series model that utilizes temporal patterns as an input to a regression equation to forecast the value at the next time step. It is a straightforward idea that can make accurate predictions for many time series problems. Linear regression models output values ​​based on linear combinations of input values [57]. This technique can be applied to time series, where input variables are used as observations at earlier time steps, so-called lagged variables [57]. In auto-regression, the regression models use data from the same input variables at an earlier time step [57]. To predict the next time step or (t + 1) based on the last two observations (t-1) and (t-2), an autoregression model can be expressed as:

2 $$\mathrm{X}(\mathrm{t}+1)={\mathrm{b}}_{0}+{\mathrm{b}}_{1}*\mathrm{X}(\mathrm{t}-1)+{\mathrm{b}}_{2}*\mathrm{X}(\mathrm{t}-2)$$

A residual error (RE) [58] is a difference between the expected and predicted values. RE generates a temporal form of information that we can model to correct existing time-series predictions [58]. Any temporal structure in the residual forecast error time series can be used as a diagnostic because it implies information that might be included in the forecast model. An ideal model leaves no structure in the residuals. A simple and effective residual model is auto-regression where some lag error values ​​are used to predict the error at the subsequent time step. Such lag errors are conjoined in a linear regression model, such as the AR model for a direct time-series analysis. Auto-regression of residual time series is called a moving average (MA) model. We created a model with autoregression to model the RE time series in a linear form. The linear model yields a weighted linear summation of lagged RE terms, and that can be expressed as:

3 $$\mathrm{Error}(\mathrm{t}+1)={\mathrm{b}}_{0}+{\mathrm{b}}_{1}*\mathrm{Error}(\mathrm{t}-1)+{\mathrm{b}}_{1}*\mathrm{Error}(\mathrm{t}-2)+..+ {\mathrm{b}}_{\mathrm{n}}*\mathrm{Error}(\mathrm{t}-\mathrm{n})$$

Our activity data shows the number of steps per minute. We converted it to a daily step count for daily step forecasting. Time-series [59, 60] data is strictly sequential; however, highly prone to non-stationarity, autocorrelation, trend, and seasonality. We used the Augmented Dicky-Fuller hypothesis test [59, 60] with autolog = ‘AIC’ and regression = ‘CT/C’ to verify the stationarity of the time-series data. We used seasonal decomposition with model = ‘additive’ or ‘multiplicative’ to analyze the data's trend, seasonality, and residual components. We converted the non-stationary data to stationary with the different transform methods. We used an autocorrelation (ACF) 2D plot to observe the lag value (X-axis) and the correlation (Y-axis) between -1 and 1, and partial autocorrelation (PCF) with limited lag value (e.g., 25, 50). ACF and PCF had been useful for parameter selection in time-series forecasting models. We have used the autoregression model with the integration of the residual error minimization technique to forecast time-series step data. We have created a lag value of 50 for the PMData datasets and 14 for the MOX2-5 datasets. We have considered an autoregression window length of five for both datasets. The steps for autoregression with residual error minimization to improve univariate time-series forecasting are described in Table 8.

**Table 8:** Steps for residual error minimization in univariate time-series forecasting

Step 1: Creation of lagged datasets
Step 2: Split data into train and test with a 60:20:20 ratio using the stratification technique
Step 3: Apply the persistence model by predicting the output value (Y) as a replica of the input value (X)
Step 4: Calculate residuals
Step 5: Model the training set residuals with a defined lag value, predict RE with the AR model, and defined window size ⋲ Z +
Step 6: Walk forward over time steps in the test dataset
Step 7: Correct forecasts with the designed model of RE using the following equation: $improved\;forecast=forecast+estimated\;\;error$
Step 8: Calculate metrics for the corrected forecasts and compare them with the forecasts without REM to observe the improvements

### Ensemble classification algorithms

This study has performed multi-class classification with standard ensemble machine learning models [61–63], such as bagging, boosting, and voting, followed by empirical comparison testing. We have used ensemble classifiers instead of deep learning classifiers because of the following reasons: convex optimization technique in gradient descent to find global minima, small amounts of training data, lesser model training time, training on a central processing unit (CPU), computationally inexpensive in terms of time and space, and transparency.

Regularizations, such as L1-norm and L2-norm have not been added to the models because of the limited set of features. Ensembles [61, 62] can give a boost to classification results in combination with several supervised models based on the approaches, such as parallel ensemble (bagging), sequential ensemble (boosting), and voting. Bagging is a bootstrap aggregation method where homogeneous weak learners are clubbed together in parallel with a deterministic averaging process to improve prediction accuracy by decreasing model variance. Random Forest (RF), Bagging Classifier, and ExtraTreesClassifier are examples of bagging ensembles and are used here for comparative performance analysis. Boosting is an incremental method in which multiple weak learners are combined sequentially over numerous iterations using a deterministic strategy to build a strong ensemble. AdaBoost (ADA) and Gradient Boosting Classifier (GB) are examples of boosting ensembles and are used here for performance comparison. Voting combines predictions based on standard statistical metrics, such as the mean median, to decide the final prediction.

To better use data, initially, we shuffled the dataset, then split the dataset into training and testing with a random state integer value. To boost the performance of the machine learning model, we used k-fold cross-validation where k >  = 5. We used Grid Search [4] hyperparameter optimization technique for machine learning model tuning for an appropriate selection of learning rate (alpha (α)) in gradient descent algorithm, and proper selection of other components, such as components, criterion, and max_depth is important for tree-based models. We executed each ensemble machine learning classification model five times and calculated their mean performance score for comparison. The step for classification is stated in Table 9.

**Table 9:** Steps for daily activity level classification

Step 1: Load activity datasets
Step 2: Encode predictive class values with one-hot encoding
Step 3: Split data into train, validation, and test with (a 60:20:20) ratio using the stratification technique
Step 4: Create classification model, M
Step 5: Compile M with value-set for optimization technique, k-fold, and metrics
Step 6: Fit model M with training data
Step 7: Improve the model with a grid-search technique
Step 8: Calculate accuracy and other classification metrics
Step 9: Select the best learning parameters as computed with the Grid Search technique
Step 10: Classify input data into respective output classes

Additionally, we have used a DummyClassifier as a simple baseline to compare against other more complex classifiers. It makes predictions that ignore the input features. We have used its strategy parameter as “most_frequent”. First, we performed predictive analysis on the imbalanced physical activity datasets and then, we carried out the same experiment on balanced datasets to verify model biases. Stratification techniques helped to ensure that the distribution of target classes is preserved in the resulting subset.

### Evaluation metrics

The performance of ML-based classification models has been evaluated against discrimination analysis. Discrimination analysis metrics are precision, recall, specificity, accuracy score, F1 score, classification report, and confusion matrix [4, 61, 62]. A confusion matrix is a 2-dimensional table (“actual” vs “predicted”), and both dimensions have “True Positives (TP)”, “False Positives (FP)”, “True Negatives (TN)”, and “False Negatives (FN)”. Equations to calculate classification metrics are:

4 $$\mathrm{Accuracy }\left(\mathrm{A}\right)=\frac{\left(\mathrm{TP}+\mathrm{TN}\right)}{\left(\mathrm{TP}+\mathrm{FP}+\mathrm{FN}+\mathrm{TN}\right)}, 0\le \frac{(\mathrm{A}) }{(100)}\le 1$$

5 $$\mathrm{Precision }(\mathrm{P})=\frac{(\mathrm{TP}) }{(\mathrm{TP}+\mathrm{FP})}$$

6 $$\mathrm{Recall}(\mathrm R)\;\mathrm{or}\;\mathrm{Sensitivity}(\mathrm S)\;\mathrm{or}\;\mathrm{True}\;\mathrm{positive}\;\mathrm{rate}=\frac{(\mathrm{TP})}{(\mathrm{TP}+\mathrm{FN})}$$

7 $$\mathrm{Specificity }(\mathrm{S}) = (1 -\mathrm{ Sensitivity}) = \frac{(\mathrm{TN}) }{(\mathrm{TN}+\mathrm{FP})}$$

8 $$\mathrm{F}1\mathrm{ score }\left(\mathrm{F}1\right)=\frac{\left(2*\mathrm{P}*\mathrm{R}\right)}{\left(\mathrm{P}+\mathrm{R}\right)}, 0\le \frac{(\mathrm{F}1) }{(100)}\le 1$$

Accuracy tells how close a measured value is to the actual one. Recall or sensitivity suggests the exact number of positive measures. Precision means how relative the measured value is to the actual one. Furthermore, we used cross-validation scores to determine overfitting and underfitting, a validation curve to determine bias vs. variance, and a learning curve to visualize the convergence status of the training score with the cross-validation score. Bias is an error due to erroneous assumptions in the learning algorithm, and variance is an error from sensitivity to small fluctuations in the training set. High bias leads to underfitting, and the high variance results in overfitting. Accuracy and F1 scores can be misleading because they do not fully account for the sizes of the four categories of the confusion matrix in the final score calculation. MCC is more informative than the F1 score and accuracy because it considers the balanced ratios of the four confusion matrix categories (for example, true positives, true negatives, false positives, and false negatives). The F1 score depends on which class is defined as a positive class. However, MCC does not depend on which class is the positive class, which has an advantage over the F1 score and avoids incorrectly defining the positive class [64–66]. The MCC can be represented as:

9 $$\mathrm{Matthew}'\mathrm s\;\mathrm{correlation}\;\mathrm{coefficient}\;\left(\mathrm{MCC}\right)=\frac{\left(\mathrm{TP}\left(\mathrm{TP}\ast\mathrm{TN}-\mathrm{FP}\ast\mathrm{FN}\right)\right)}{\sqrt{\left(\mathrm{TP}+\mathrm{FP}\right)\left(\mathrm{TP}+\mathrm{FN}\right)\left(\mathrm{TN}+\mathrm{FP}\right)\left(\mathrm{TN}+\mathrm{FN}\right)}},-1\leq\frac{(\mathrm{MCC})}{(100)}\leq+1$$

The performance of each time-series forecasting model has been evaluated with root mean squared error (RMSE). MSE informs how close the regression line is to a set of points. It calculates “errors” from the points to the regression line and squares them to eliminate negative signs. The squared root of MSE is called RMSE, which gives more weight to a significant difference with no bias [4, 60]. We used other metrics such as forecast bias (FB), RSD, and model execution time (in seconds or sec.). FB can be positive or negative. A non-zero mean prediction error value implies the tendency of the model to predict too high (negative error) or too small (positive error). Thus, the mean forecast error is called FB. If forecast error = 0, the forecast has no error for that prediction. Overprediction if the prediction variance < 0, and the model is unbiased if the prediction variance ≈ 0 [60].

Our proposed ontology model has been evaluated against reasoning time and SPARQL query execution time [5]. Protégé provides a list of reasoners, such as HermiT, Pellet, Fact +  + , RacerPro, and KAON2, to check the logical and structural consistencies [5, 43]. We compared mean reasoning time and selected the best reasoner for our ontology. We have captured the SPARQL query execution time in Protégé. Furthermore, we cross-verified the execution time of the ontology in the Jena Fuseki server [5].

### Probabilistic interval prediction

In predictive inference, a prediction interval is an estimate of an interval containing future observations with some probability, based on what has already been observed. Prediction intervals are often used in prediction analysis. In this study, we have used the concept of step forecasting. The prediction interval which gives the gap to maintain a specific probability value, can be written as [67, 68]:

10 $${\mathrm{Y}}_{\mathrm{T}+\mathrm{h}|\mathrm{T}}\pm \mathrm{c}*\mathrm{\sigma h}$$

“c” changes with coverage probability. In one-step interval prediction “c” is 1.28 (80% prediction interval where forecast errors are normally distributed). “σ_h_” is the estimation of the residual standard deviation (RSD) in the h-step forecast distribution (h>0). RSD is used to statistically describe the difference in the standard deviation of observed values versus the standard deviations of estimated values. We have used the Naïve forecast method to statistically derive “σ_h_”.

### Validation study

The validation part explains the performances of the best-performing classification models on both the balanced and imbalanced datasets and the verification of the intended personalized recommendation generation and its appropriate visualization.

#### Verification of the classifiers

To verify the performance of the classifiers in public Fitbit and MOX2-5 datasets, we have the following two visualization approaches: (a.) Validation curve: it is an essential diagnostic tool that shows the sensitivity between changes in the accuracy of an ML model and changes in specific model parameters. A validation curve is usually drawn between some model parameters and the model's score. There are two curves in the validation curve—one for the training set score and one for the cross-validation score. Validation curves evaluate existing models based on hyperparameters, and (b.) Learning curve: it shows the estimator's validation and training scores for different numbers of training samples. It is a tool to see how much we benefit from adding more training data and whether the estimator suffers more from variance or bias errors.

#### Verification of personalized recommendation generation and visualization

For personalized recommendation generation in the eCoach prototype system, we have maintained individual preferences to understand personal interests (e.g., Table 6). Preference data are stored in the KB. Participants can view and update their preference data in the eCoach mobile app. To determine the weekly score of personal goal achievement, we have summed up the daily activity score, and the measure of the daily activity score is mentioned in Table 2.

## Results

### Experimental setup

We used Python 3.8.5 libraries, such as pandas (v. 1.1.3), NumPy (v. 1.21.2), SciPy (v. 1.5.2), Matplotlib (v. 3.3.2), Seaborn (v. 0.11.0), Plotly (v. 5.2.1), scikit-learn (v. 0.24.2), Statsmodels (v. 0.13.2), and Graph Viz (v. 2.49.1) to process data and build the AI models. We have set up the Python environment in the Windows 10 operating system using Anaconda distribution and used the Jupyter Notebook v. 6.4.5 for the development, model analysis, and data visualization. The targeted system consists of 16GB RAM and 64-bit architecture. We used the Protégé 5.x open-source editor for ontology design, implementation, SPARQL query processing, and visualization.

### Experimental results

**Fig. 7:**
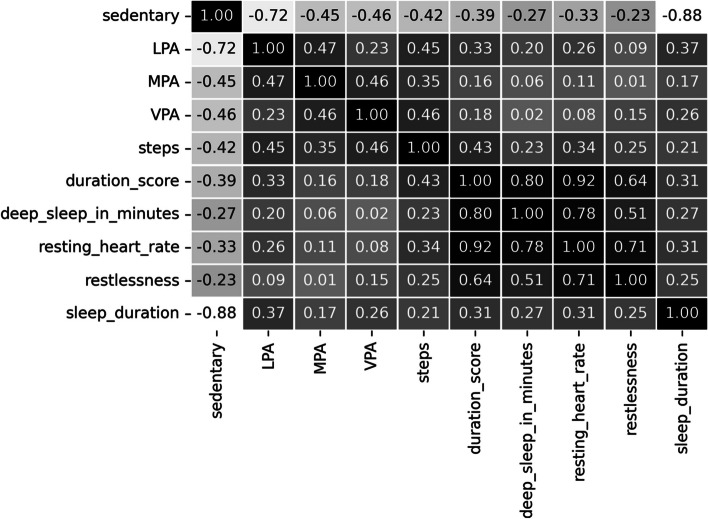
Correlation matrix for the public PMData datasets

**Fig. 8:**
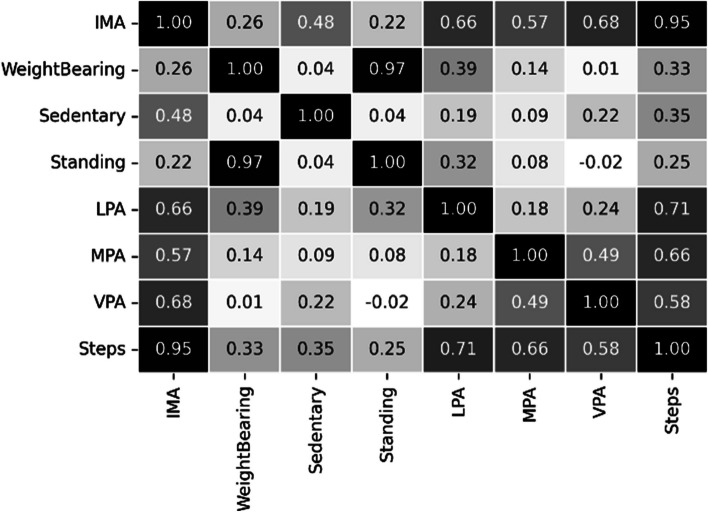
Correlation matrix for the private MOX2-5 datasets

The correlation matrix of the selected features from the PMData and MOX2-5 datasets has been captured in Figs. 7 and 8. “Step” has been found to be an essential feature in both activity datasets. We have prepared our final feature set based on the outcome of the feature correlation score. The feature “calorie” is not related to the context of this study. Therefore, the final feature set can be written as:

11 $$\mathrm{Final}\;\mathrm{feature}-\mathrm{set}=(\mathrm{FS}-1\bigcap\mathrm{FS}-2)=\{\mathrm{sedentary},\;\mathrm{LPA},\;\mathrm{MPA},\;\mathrm{VPA},\;\mathrm{steps}\}$$

**Fig. 9:**
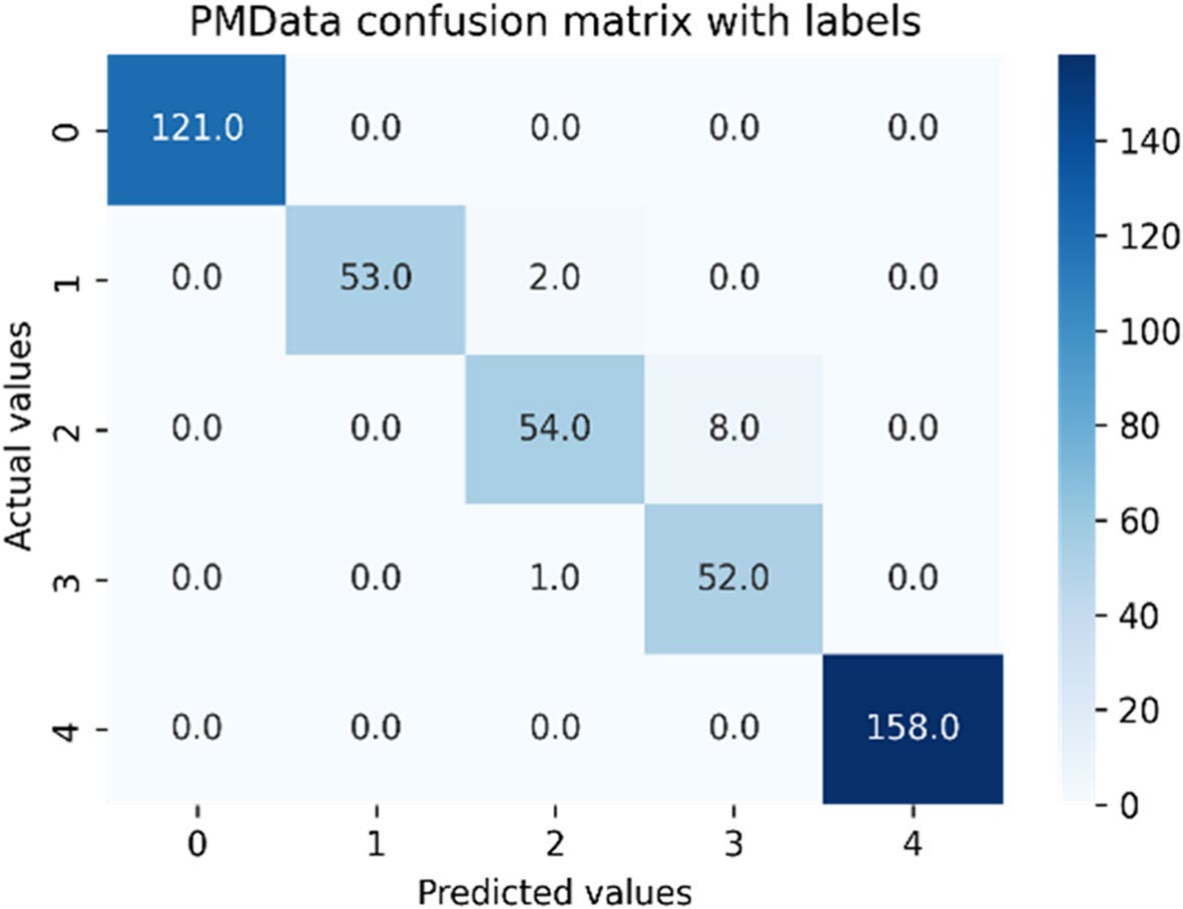
Confusion matrix for the public original PMData datasets

**Fig. 10:**
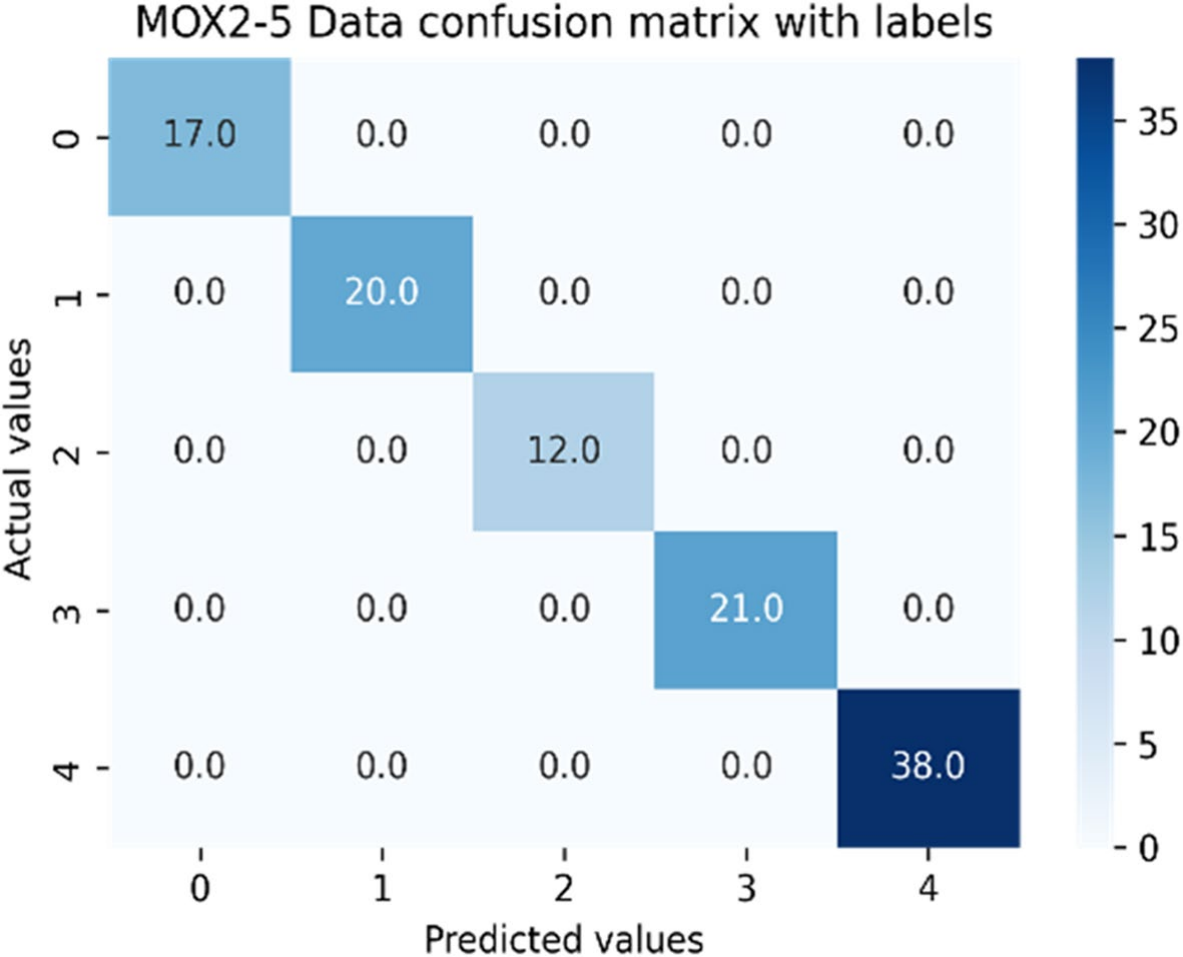
Confusion matrix for the private original MOX2-5 datasets

The performance of standard ensemble models depends on the nature of datasets used for a particular case study, under a defined setting. Therefore, we have tried different standard ensemble classifiers with unique settings using the Grid-Search method. The comparative analyses between considered ensemble classifiers for both datasets have been captured in Tables 10 and 11. The GB classifier outperformed other classifiers on both datasets to classify daily human activity levels into five activity-level classes. Therefore, GB has been successful in combining weak learners to deliver improved classification accuracy. The resultant confusion matrices for both datasets against the GB classifier have been depicted in Figs. 9 and 10. {'learning_rate': 0.1, 'max_depth': 3, 'n_estimators': 100} have been the best tree-specific hyperparameters for the GB classifier to affect each individual tree in the model as obtained with the Grid Search for PMData and MOX2-5 datasets, respectively. The training vs. testing, validation, and learning curves for the Gradient Boosting classifier used in both datasets are depicted in Additional file 3: Appendix A.6 – Appendix A.8.

**Table 10:** Performance of the machine learning classifiers for public original Fitbit datasets

ML classifier models	Mean accuracy	Precision	Recall	F1	MCC
RF	98.00	98.00	98.00	97.29	96.60
Bagging	98.00	98.00	98.00	97.24	96.30
ExtraTreesClassifier	97.00	97.00	97.00	97.16	95.30
ADA	91.00	88.00	89.00	88.19	84.98
GB	**98.00**	**98.00**	**98.00**	**97.86**	**96.78**
Voting	96.00	96.00	96.00	96.21	94.99
DummyClassifier	35.18	12.00	35.00	18.00	00.00

**Table 11:** Performance of the machine learning classifiers for private original MOX2-5 datasets

ML classifier models	Mean accuracy	Precision	Recall	F1	MCC
RF	99.00	99.00	99.00	99.07	98.80
Bagging	99.00	99.00	99.00	99.20	98.70
ExtraTreesClassifier	99.00	99.00	99.00	99.07	98.85
ADA	59.00	70.00	62.00	70.30	67.17
GB	**99.00**	**99.00**	**99.00**	**99.50**	**99.00**
Voting	95.00	93.00	92.00	92.50	90.80
DummyClassifier	27.31	27.00	27.00	27.00	00.00

**Fig. 11:**
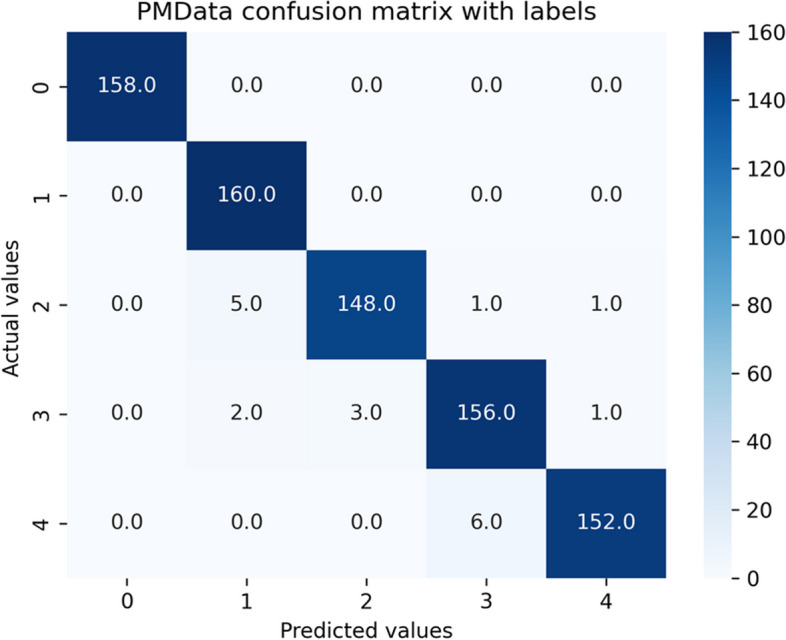
Confusion matrix for the public *balanced* PMData datasets

**Fig. 12:**
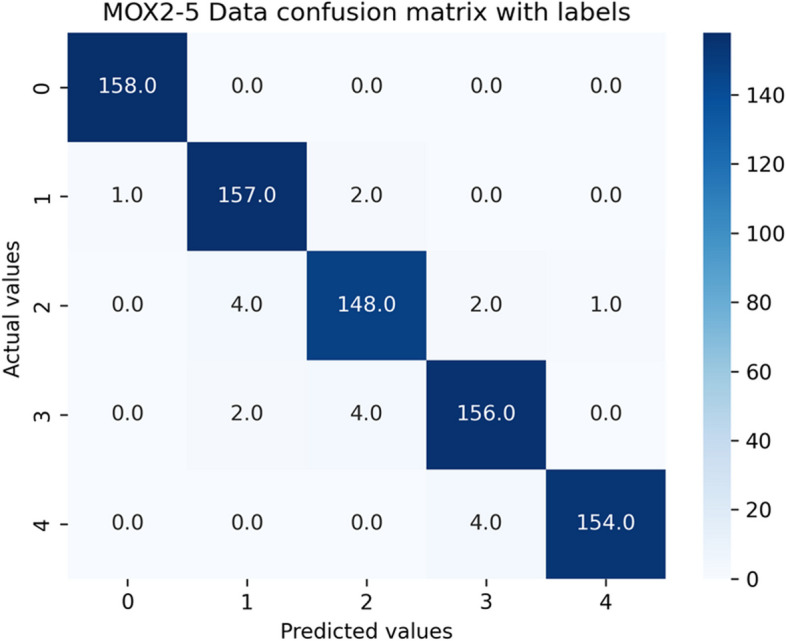
Confusion matrix for the private *balanced* MOX2-5 datasets

We repeated the predictive analysis on PMData and MOX2-5 datasets using the Ensemble Classifiers after making their distributions balanced based on the local density of minority samples, with the well-known ADASYN sampling algorithm to verify if data balancing is improving the model performance and fairness or not. ADASYN augmented PMData by 76.6% (3964–2244 = 1720) and MOX2-5 by 79.9% (970–539 = 431). The resultant confusion matrices for both balanced datasets against the GB classifier have been depicted in Figs. 11 and 12. {'learning_rate': 0.1, 'max_depth': 3, 'n_estimators': 100} have been the best tree-specific hyperparameters for the GB classifier to affect each individual tree in the model as obtained with the Grid Search for balanced PMData and balanced MOX2-5 datasets, respectively. The comparative analyses between considered ensemble classifiers for both the balanced datasets have been captured in Tables 12 and 13.

**Table 12:** Performance of the machine learning classifiers for public *balanced* Fitbit datasets

ML classifier models	Mean accuracy	Precision	Recall	F1	MCC
RF	97.80	97.00	98.00	97.80	95.60
Bagging	97.60	97.00	98.00	97.60	95.30
ExtraTreesClassifier	98.20	98.00	98.00	98.20	96.30
ADA	81.50	81.00	82.00	82.50	78.00
GB	**98.30**	**98.00**	**98.00**	**97.30**	**96.40**
Voting	90.50	91.00	91.00	90.50	88.00
DummyClassifier	20.50	21.00	21.00	20.50	00.00

**Table 13:** Performance of the machine learning classifiers for private *balanced* MOX2-5 datasets

ML classifier models	Mean accuracy	Precision	Recall	F1	MCC
RF	99.50	99.00	99.00	99.50	98.00
Bagging	99.50	99.00	99.00	99.50	98.00
ExtraTreesClassifier	99.50	99.00	99.00	99.50	98.00
ADA	76.50	76.00	77.00	76.50	72.07
GB	**99.80**	**99.00**	**99.00**	**99.80**	**99.00**
Voting	75.00	75.00	75.00	75.00	72.80
DummyClassifier	20.72	20.70	20.70	20.72	00.00

Tables 14 and 15 have shown the performance comparison between the autoregression model with the residual error minimization technique and the traditional autoregression model without the residual error minimization technique. The residual error minimization technique has improved the model's performance according to the prediction outcomes. As an example, the ACF, PCF, and autoregression with residual error minimization prediction plots for the participant-1 or P-1 from the PMData datasets are depicted in Additional file 3: Appendix A.9 and Appendix A.10.

**Table 14:** Mean prediction performance on original PMData datasets

**Models**	**RMSE**	**| FB |**	**RSD**	**ET (sec.)**
Autoregression with residual error minimization	5936.50	223.40	1475.60	144.00
Autoregression without residual error minimization	6590.00	252.00	1863.00	149.00

**Table 15:** Mean prediction performance on original MOX2-5 datasets

**Models**	**RMSE**	**| FB |**	**RSD**	**ET (sec.)**
Autoregression with residual error minimization	5936.50	223.40	1475.60	144.00
Autoregression without residual error minimization	6590.00	252.00	1863.00	149.00

**Fig. 13:**
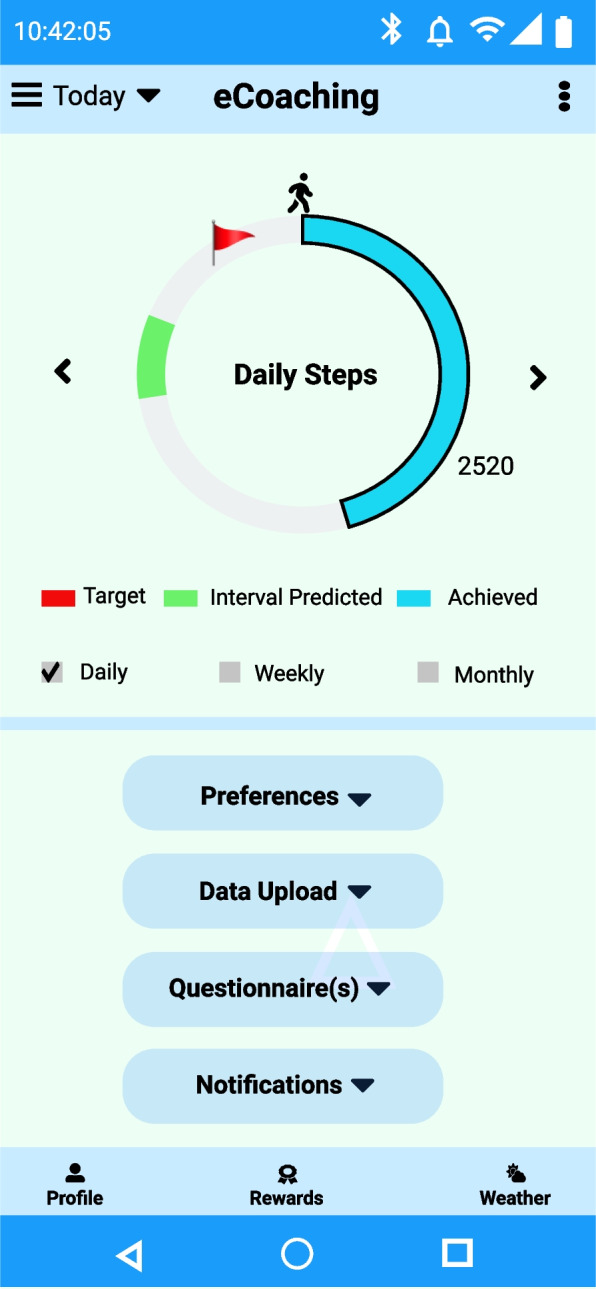
Visualization of daily step count, target step count, and predicted interval

Interval prediction can be a meaningful representation of temporal daily step counts. Therefore, we have used autoregression with the residual error minimization for the next seven days' step forecast for participants’ data from the PMData datasets, based on its temporal step data analysis. As an example, we have calculated the RSD value ≈ 2600.0 for the step data of P-1. Using the informed probabilistic Naïve interval prediction method, we have shown a direction to calculate the 1-step interval prediction of activity steps on top of the point prediction (see Table 16). An approach to present the interval step prediction in the ActieCoach mobile app to motivate individuals for their personal activity monitoring to reach activity goals has been implemented and is shown in Fig. 13. A similar process can be accomplished for other participants in the PMData and MOX2-5 datasets.

**Table 16:** Step and Interval prediction for Week-X for P-1 in PMData datasets

Week-X	Predicted step points	80% interval step prediction c = 1.28, σ_h_ = 2600.00
Day-1	7369	7369 ± 3328
Day-2	8879	8879 ± 3328
Day-3	8202	8202 ± 3328
Day-4	7557	7557 ± 3328
Day-5	10,199	10,199 ± 3328
Day-6	9819	9819 ± 3328
Day-7	7426	7426 ± 3328

We have used the “OWL_MEM_MICRO_RULE_INF” specification (OWL full) to read the ontology in Jena in the “TTL” format and approximated the reading time to 1.0–1.5 s. Moreover, we used “In-memory” storage, “optimized rule-based reasoner OWL rules” and the Jena framework to query the ontology class, ontology, predicate, subject, and object of each sentence in < 1.0 s, < 2.0 s, and < 2.0 s, respectively. We have implemented the RDF interface provided by Jena to persist the modeled ontology and its instances in the TDB and load them back for further processing. The reasoning time of the ontology has been evaluated against the following reasoners: HermiT, Pellet, Fact +  + , RacerPro, and KAON2, and the corresponding reasoning time has been captured in Table 17. The HermiT reasoner has performed the best.

**Table 17:** Performance analysis of different reasoners available in Protégé

Reasoner(s)	Average reasoning time (sec.)
HermiT	1–1.5 s
Pellet	2–3.5 s
Fact + +	2.5–3.5 s
RacerPro	1.5–2.5 s
KAON2	2.5–3.5 s

## Discussion

The discussion section consists of key findings from this study, conceptual implementation of the proposed algorithm for recommendation generation, relevance of this study towards a sustainable society, as well as an exploration of its limitations and potential future directions.

### Key findings

In Fig. 1, the TDB database functioned as a KB. All the messages as described in Additional file 3: Appendix A.2 were saved in the KB. The recommendation generation module (see Fig. 1) retrieved these messages during tailored recommendation generation based on a SPARQL query execution plan, followed by applying the rules in Additional file 3: Appendix A.3. The rules are also kept in the KB. Both the asserted and inferred information attained from the reasoning method had been effective in determining the most appropriate recommendation message. We generated personalized activity recommendations according to the semantic rules to improve individual physical activity to meet activity goals. We executed the semantic rules and used the Jena ARQ engine to run relevant SPARQL queries on the applied datasets. Several SPARQL queries are provided in Additional file 3: Appendix A.11 as an example, and their results need to be combined to create personalized recommendations to meet the eCoaching requirements.

Furthermore, this sub-section describes the overall process of daily and weekly score determination for activity performance, goal verification, recommendation generation, and its visualization on the eCoach app as a push notification. To verify the personalized recommendation generation in participant data (i.e., MOX2-5 data), we have divided each participant’s activity days (n) into the following two parts – a. (n-7) days window for training the best-performing ensemble classifier model, and b. the remaining seven days windows for testing with an incremental learning approach. The incremental learning approach has helped in activity classification on the day-(n + 1) based on model training with personal activity data up to day-n. We repeated the same incremental process until the goal periods were completed (here, we assumed it was a window of 7 days). Moreover, we have used three standard emojis in recommendation visualization to motivate participants based on their weekly goal accomplishment (well done or good work (😊), up-to-the-mark or satisfactory performance (😐), and improved performance (☹)).

**Fig. 14:**
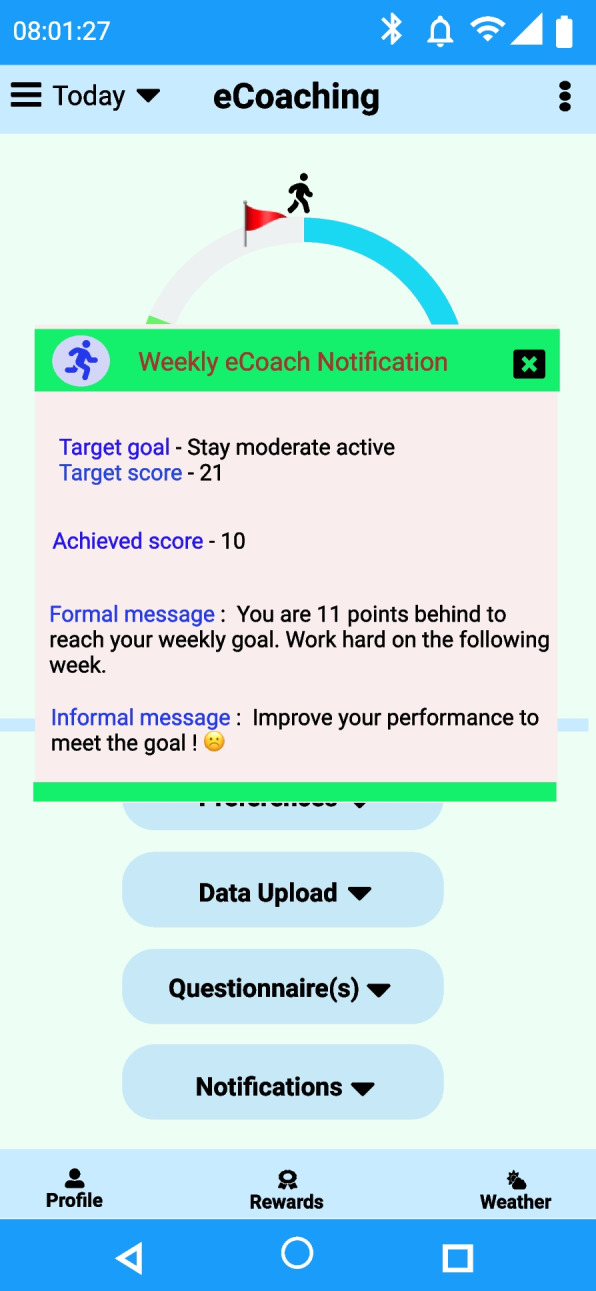
Weekly recommendation generation as a text (e.g., push notification) for P-1

For example, in the last week, participant P-1 from the MOX2-5 datasets (see Additional file 2: Appendix B for the dataset) failed to achieve WHO’s generic activity goal to stay medium active for a week. Therefore, based on the semantic rule, he received recommendation message A-17. Based on the step forecast results with our proposed autoregression with residual error minimization model, P-1 received recommendation message A-13 for the following week. We have shown the overall daily and weekly recommendation generation process and its meaningful presentation for a single participant (P-1) from the private MOX2-5 datasets (see Table 18) collected for this study. A sample recommendation generation screen has been captured in Fig. 14. However, the same approach can be applied to other participants (P-2 to P-16) in MOX2-5 datasets.

**Table 18:** Activity classification and personalized recommendation generation for P-1 in the MOX2-5 dataset

Days	The best performing classifier model(s) used in transfer learning	Actual activity level on day-n	Activity level predicted on day-n after incremental learning	Daily achieved score predicted	Propositional variable
Day-1	GB classifier	Low Active	Low Active	1	A-2, A-6, A-8, A-10, A-15
Day-2		Low Active	Low Active	1	A-2, A-6, A-8, A-10, A-15
Day-3		Medium Active	Medium Active	3	A-4, A-6, A-9, A-11, A-14
Day-4		Active	Active	2	A-3, A-6, A-8, A-10, A-15
Day-5		Low Active	Low Active	1	A-2, A-6, A-8, A-10, A-15
Day-6		Low Active	Low Active	1	A-2, A-6, A-8, A-10, A-15
Day-7		Low Active	Low Active	1	A-2, A-6, A-8, A-10, A-15
Weekly Score	Achieved = ∑Daily_achieved_score_predicted = (1 + 1 + 3 + 2 + 1 + 1 + 1) = 10				-
Prediction accuracy	∑Daily_achieved_score_actual—∑Daily_achieved_score_predicted = 0				-
Difference	Defined_goal_score—Achieved = (21 – 10) = 11				-
Weekly Recommendation	***Formal message—****“You are 11 points behind to reach your weekly goal. Work hard on the following week.*” ***Informal message –**** “Improve your performance to meet the goal!* ☹*”*				A-17, A-13

From the classification and forecasting results on both datasets, we can confirm that we have successfully designed and developed AI models in ActieCoach for time-series prediction and activity-level classifications. According to the results, an increased volume of MOX2-5 datasets will improve individual model stability and learning. In both datasets, model loss for training and testing converges. Future step prediction for individuals combined with the statistical approaches (e.g., weekly activity weighted means and/or standard deviation calculation) can be a useful direction for generating personalized recommendations.

The average SPARQL queries' execution time has been captured between 0.1 s – and 0.3 s (sec). The semantic rules described in Additional file 3: Appendix A.3 represent the logic behind personalized recommendation message generation. The rule-based binary reasoning (If ➔ 1, else ➔ 0) helps to explain the formation of a personal activity recommendation message. A complete data-driven approach to personalized recommendation generation in healthcare is critical due to false-positive scenarios. Therefore, prediction modeling followed by an annotated ruleset can produce more value for personalized health recommendations.

Overall, this study rigorously focuses on automizing the personalized activity recommendation generation with an AI, personal preference information, adjustable rule base, and their integration with a semantic network for reasoning and meaningful querying for personalized recommendations. Personalization is important in health recommendations to understand the user context and perspective. Therefore, health recommendation algorithms are contextually different from traditional user or item-based recommendation algorithms which are well-accepted in the commercial domains. In Table 16, we have accomplished the efficiency of applying our proposed hybrid recommendation algorithm in activity eCoaching in an empirical way. The *Daily achieved score predicted* column in Table 16 describes the reason behind the recommendation generation in the *Propositional variable* column. Such a study in eCoaching has not been conducted according to the existing literature. Therefore, we have restricted Table 1 to a qualitative comparison instead of an empirical comparison.

### Relevance

Our proposed physical activity eCoaching is intended to facilitate long-term behavior alteration and reduction of a sedentary lifestyle. Constantly providing healthy lifestyle assistance, self-management instructions for healthy lifestyle goal-management, and individualized counseling, eCoaches facilitate the development of healthy behaviors that can be maintained following a specific program or intervention. Overall, activity eCoaching has a significant impact on the contribution to the United Nations' Sustainable Development Goals (SDG) 3 [69], which is concerned with ensuring healthy lifestyles and promoting well-being across all ages. Through technology, personalization, and behavioral alteration, our activity eCoaching may align with the objectives of SDG-3 by promoting physical fitness, preventing diseases, empowering individuals, and contributing to the overall goal of having healthy lives and enhancing well-being for all.

### Limitations and future scope

Our used datasets are small, and we thought they might be biased. High bias leads to model underfit. Therefore, we have used the MCC metric to understand the ensemble models' performance in a better way. However, more data is required to train and test the classifier models. The data balancing technique is popular in predictive analysis to verify bias and fairness in classification models. Thus, we have restricted our experimental analysis to imbalanced and balanced datasets for classification or predictive analysis, instead of regression analysis. The comparative predictive analysis (Tables 10, 11, 12, 13 and 14) of both imbalanced and balanced datasets reveals that models are not biased, and data balancing can improve model performances. Our implemented approach toward the collection of personalized physical activity data based on consent, test for model biases, fairness, and scalability with metrics (e.g., MCC) and data balancing algorithm, data governance following the GDPR guidelines, steps toward the handling of growing data with automatic and continuous model training-validation-testing has made the eCoaching approach ethical and trustworthy.

Our proposed approach in Fig. 1 gives design flexibility with a modular design approach to deal with other deep learning classifiers and forecast algorithms. In that case, we need to update the prediction and classification algorithms in the data processing and activity prediction module. The ontology tree supports branching and pruning to integrate new ideas. The knowledge base and the recommendation message table can grow or shrink based on the preferences and study requirements. This design approach can also support other activity sensors (e.g., Actigraph). This study proves an integrated concept for tailored hybrid recommendation generation in activity eCoaching. We can include deep learning-based classification and prediction for performance comparison, stability analysis, and more activity attribute support with the growing activity data. We will improve the recommendation generation with other approaches, such as clustering and similarity score, and calculate and compare activity intensity across different weeks using the statistical and community-based heuristic methods. A person can receive multiple recommendation messages, and the solution can be enhanced with a meta-heuristic approach to select an optimal set of recommendations from a feasible recommendation set.

In authentic coaching, to attain a weekly or monthly goal, as a part of continuous monitoring, the eCoach module will generate personalized recommendations on time, based on the activity outcome on each day, followed by a predictive analysis to achieve the weekly goal. Moreover, this is not authentic coaching but conceptual modeling with AI technology and semantics. To evaluate the practical effectiveness of the concept, further study is needed on a cluster of controlled trials.

## Conclusion

This study has shown a direction to use standard AI technology, ethics, data governance, personal preferences, and semantic ontology to design and develop an intelligent eCoach system with semantic knowledge representation to generate automatic, meaningful, contextual, and personalized activity recommendations to attain personal activity goals. To improve individual physical activity levels with wearable activity sensors and digital activity trackers, eCoach features can be encouraging. The concept of univariate time-series forecasting exists; its application with an ontology and interval prediction for activity eCoaching is novel. Furthermore, in this study, we have proved the hypothesis that an auto-regression model with the residual error minimization technique can produce better performance than an auto-regression model without the residual error minimization technique. Moreover, this study has presented a detailed analysis of different standard ensemble classifiers on balanced and imbalanced physical activity data, elaborated classification results, investigated for bias, and ethical aspects of AI, and thereby generated understandable and meaningful personalized activity recommendation generation with semantic rules and SPARQL query execution. We will extend this study with the integration of concepts such as activity density and clustering to make eCoach recommendations more realistic and evidence-based on a group of controlled trials.

### Supplementary Information

**Additional file 1.**

**Additional file 2:** **Appendix B: **StaRI checklist for completion.

**Additional file 3: Appendix A.1: Table A.1.** provides a high-level description of the used terminologies or parameters in this study.** Appendix A.2: Table A.2.** provides in-context propositional variables and corresponding physical activity recommendation messages.** Appendix A.3: Table A.3.** describes recommendation conditions, semantic rules based on the propositional variables, and execution criteria.** Appendix A.4: Table A.4.** provides details of the attributes of the MOX2-5 physical activity datasets.** Appendix A.5: Table A.5.** provides in detail participant characteristics (such as factors) and the statistical estimation of the used factors (such as mean, standard deviation, minimum, and maximum values) for the 16 participants who used the MOX2-5 activity sensor.** Appendix A.6: Figure A.6.** compares the training vs. testing curve of the original PMData and MOX2-5 datasets against the best-performing GB classifier.** Appendix A.7: Figure A.7.** compares the learning curve of the original PMData and MOX2-5 datasets against the best-performing GB classifier.** Appendix A.8: Figure A.8.** compares the validation curve of the original PMData and MOX2-5 datasets against the best-performing GB classifier.** Appendix A.9: Figure A.9.** depicts ACF and PCF plotting for plot for the participant-1 (P-1) from the original PMData dataset as an example for the regression analysis.** Appendix A.10: Figure A.10.** depicts the prediction comparison between the autoregression model with residual error minimization (predicted) and the autoregression model without residual error minimization for the P-1 from the original PMData dataset as an example.** Appendix A.11: Textbox A.11.** describes all the used and selected list of Ontology Schema, and SPARQL queries as a part of personalized recommendation generation.

## Data Availability

All the data used or produced in this study are either in the main text or in the supplementary files. The corresponding author AC can be contacted for the datasets and codebase.

## Abbreviations

ACF: Autocorrelation
ADASYN: Adaptive Synthetic
ADA: ADA Boost
AI: Artificial Intelligence
eCoach: Electronic Coaching
ET: Execution Time
FB: Forecast Bias
GDPR: General Data Protection Regulation
GB: Gradient Boost
IMA: Activity Intensity
KB: Knowledge Base
LPA: Low Physical Activity
MCC: Matthews Correlation Coefficient
MPA: Medium Physical Activity
NSD: Norwegian Centre for Research Data
OWL: Semantic Web Language
PCA: Principal Component Analysis
PCF: Partial Autocorrelation
RDF: Resources Description Framework
REK: Regional Committees for Medical and Health Research Ethics
RF: Random Forest
RFE: Recursive Feature Elimination
RSD: Residual Standard Deviation
SMOTE: Synthetic Minority Oversampling Technique
SPARQL: SPARQL Protocol and RDF Query Language
SWRL: Semantic Web Rule Language
TDB: Tuple Database
VPA: Vigorous Physical Activity
WHO: World Health Organization
