# Supplementary material for: AI and semantic ontology for personalized activity eCoaching in healthy lifestyle recommendations: a meta-heuristic approach
Source: BMC Med Inform Decis Mak. 2023 Dec 1;23:278. doi: 10.1186/s12911-023-02364-4 (PMC10693173; doi:10.1186/s12911-023-02364-4)
Supplement: Supplementary file 3 — Additional file 3: Appendix A.1: Table A.1. provides a high-level description of the used terminologies or parameters in this study. Appendix A.2: Table A.2. provides in-context propositional variables and corresponding physical activity recommendation messages. Appendix A.3: Table A.3. describes recommendation conditions, semantic rules based on the propositional variables, and execution criteria. Appendix A.4: Table A.4. provides details of the attributes of the MOX2-5 physical activity datasets. Appendix A.5: Table A.5. provides in detail participant characteristics (such as factors) and the statistical estimation of the used factors (such as mean, standard deviation, minimum, and maximum values) for the 16 participants who used the MOX2-5 activity sensor. Appendix A.6: Figure A.6. compares the training vs. testing curve of the original PMData and MOX2-5 datasets against the best-performing GB classifier. Appendix A.7: Figure A.7. compares the learning curve of the original PMData and MOX2-5 datasets against the best-performing GB classifier. Appendix A.8: Figure A.8. compares the validation curve of the original PMData and MOX2-5 datasets against the best-performing GB classifier. Appendix A.9: Figure A.9. depicts ACF and PCF plotting for plot for the participant-1 (P-1) from the original PMData dataset as an example for the regression analysis. Appendix A.10: Figure A.10. depicts the prediction comparison between the autoregression model with residual error minimization (predicted) and the autoregression model without residual error minimization for the P-1 from the original PMData dataset as an example. Appendix A.11: Textbox A.11. describes all the used and selected list of Ontology Schema, and SPARQL queries as a part of personalized recommendation generation. [file 12911_2023_2364_MOESM3_ESM.docx]

Appendix A.1.

Table A.1: A high-level description of the used terminologies.

| **Parameters** | **Description** |
| --- | --- |
| Hybrid recommendation generation | A hybrid recommendation system integrates multiple recommendation algorithms as a single unit. Solely data-driven or rule-based recommendation systems have several disadvantages in healthcare. In this study, we have adopted a hybrid recommendation architecture where data-driven prediction results are combined with a rule base. |
| Semantic Modeling with ontology and ontology tree in decision making | Ontology supports flexibility in solving real-world modeling and knowledge representation problems. An ontology has a tree-like hierarchical structure (О_h_) with the following properties. An ontology О is defined as a tuple Ω = {Ć, R}, where Ć is the set of concepts and R is a set of relations:  L = Levels (О_h_) = Total number of levels in the ontology hierarchy, 0 ≤ n ≤ L, where n⋲ Z^+^ and n=0 represents the root node  C_n,j_ = a model classifying О at a level n; where, j ⋲ {0, 1,..\|C_n_\|}  \|C\| = Number of instances classified as class C  E = Edge (C_n,j_, C_n-1, k_) = edge between node C_n,j_ and its parent node C_n-1, k_  Ontologies have specific benefits in modeling concepts, data, and recommendation generation. It helps interpret which recommendation message to generate using a binary tree-like structure (if-then or if-then-else conditional statement). |
| Probabilistic interval prediction | Prediction intervals provide a way to quantify the uncertainty of a single future observation, assuming that the underlying distribution is normal. In PIP, we express uncertainty in predictions through probability distributions. The point forecast is the average of this distribution. It describes the probability of observing possible future values ​​with the fitted model. |
| Observation with activity sensor or tracker | Activity trackers record individual movements throughout the day. These sensors measure many parameters such as acceleration (e.g., steps taken during the day, distance traveled, duration of the activity, whether you are reaching your daily goals), frequency, duration, intensity, and patterns of individual movements. Then they are passed through Algorithms to do some guesswork to make sense of all the readings the data provides. Popular activity trackers are MOX2-5, Fitbit, Actigraph, Xiaomi Mi Band, Huawei Watch, Apple Watch, Samsung watch, and AS Series (97, 98, 99). Wearable activity trackers are connected to the mobile app via Bluetooth short-range data transfer medium. |
| Preference settings | Preferences can be system-defined or user-defined (or generic). Preferences can be three types: goal setting, response type for intuitive coaching, and interaction type. Preferences settings are adequate for customizing tailored recommendation generation. Preference settings define the nature of goals, kind of response (e.g., direct vs. motivational, generic vs. personalized), mode (e.g., style, graph), frequency (e.g., hourly, quarterly, twice a day, once a day), and medium (e.g., audio, video, voice, text) of the interaction with an eCoach. |
| Logical recommendation generation | Interpretability makes identifying the cause-and-effect relationships between data input and data output easier. A rule base may help interpret which recommendations to follow and under which binary condition, and in this regard, ontology can be promising. In ontology, the analytical and structural presentation of knowledge, hierarchical model structuring (e.g., class, sub-class, object and data properties, axioms), and inferred knowledge generation with reasoners can solve interpretability problems in decision-making. |
| SPARQL | SPARQL Protocol and RDF Query Language is a query language used to query data stored in RDF (Resource Description Framework) format. RDF is a data format used to display information on the Semantic Web. |
| Hermit reasoner | The Hermit reasoner is a popular reasoner designed specifically for ontologies expressed in the Web Ontology Language (OWL). OWL is a language for representing and proving knowledge in a machine-understandable way. It can create rich ontologies to describe classes, attributes, and relationships between entities in different domains. The main function of the Hermit inference engine is to perform various reasoning tasks based on OWL ontology. These tasks include consistency checking, inferring subclass relationships, and categorizing individuals based on the defined classes and properties, realization, and deducing properties and relationships between entities based on the asserted information. |
| Web Ontology Language (OWL) | OWL is a part of the Semantic Web technology. It is a language for creating ontologies that describe relationships between entities in a domain. |

Appendix A.2.

Table A.2: Propositional variable and corresponding recommendation messages.

| **Type** | **Propositional variable** | **Description** |
| --- | --- | --- |
| A-1 | Sedentary | Please continue a light activity (e.g., sports 1-3 days/week, a walking goal of 5,000 to 7,499 steps/ day) |
| A-2 | Low_physically_active | Please continue more activity (e.g., sports 3-5 days/week, a walking goal of 7,500 to 9,999 steps/ day) OR do at least 150-300 minutes (2.5 – 5 hours) of moderate-intensity aerobic exercise; or at least 75-150 minutes of high-intensity aerobic exercise or perform an equivalent combination of moderate and high-intensity activities within a week to stay active |
| A-3 | Physically_active | Please continue the same or more activity based on your goal (e.g., sports 3-5 days/week, a walking goal of 7,500 to 9,000 steps/ day) |
| A-4 | Moderate_physically_active | Please continue the same or more activity based on your goal (e.g., sports 3-5 days/week, a walking goal of 10,000 to 12,499 steps/ day) |
| A-5 | Vigorous_physically_active | Please continue the same or more activity based on your goal (e.g., sports 5+ days/week, a walking goal of 12,500+ steps/ day) |
| A-6 | Sedentary_hour_negative | Please be active for z hr. more as today you were z hr. more sedentary beyond your goal. |
| A-7 | Sedentary_hour_positive | You were very active today and z hr. less sedentary; therefore, you can take that hr. of rest tomorrow. |
| A-8 | Steps_negative | Please continue x steps more tomorrow to achieve your weekly goal of x1 steps. |
| A-9 | Steps_positive | You have performed extra x steps today beyond your goal; therefore, you can do x steps less tomorrow or you can carry out the same pace. You are x1 step behind to achieve your weekly goal (OR) congratulations! You have achieved your weekly target. |
| A-10 | Activity_minute_negative | Please continue more activity of n minutes tomorrow to achieve n1 mins. of weekly goals. |
| A-11 | Activity_minute_positive | You have performed extra m minutes of activity today beyond your goal; therefore, you can be m mins. of less highly active tomorrow or you can carry out the same pace. You are n1 mins. behind to achieve your weekly goal (OR) congratulations! You have achieved your weekly target. |
| A-12 | Step_forecast_trend_postive | Based on your weekly step forecast trend in this Week-N you can achieve the step goal. |
| A-13 | Step_forecast_trend_negative | Based on your weekly step forecast trend in this Week-N you cannot achieve the step goal. On Week-XX and Week-XY weeks, you were very active. Please try to follow similar activity patterns. |
| A-14 | Daily_Goal_achieved | Good work. Please keep it up tomorrow. You are active and completed the goal for today.  Overview:  You have performed x steps today.  You slept y hrs.  You were sedentary for z hrs.  You were m minutes of medium active.  You were n minutes of highly active. |
| A-15 | Daily_Goal_not_achieved | You must improve to meet the daily goal. Please stay active tomorrow.  Overview:  You have performed x steps today.  You slept y hrs.  You were sedentary for z hrs.  You were m minutes of medium active.  You were n minutes of highly active. |
| A-16 | Weekly_Goal_achieved | Good work. Please keep it up next week. You are active and completed the goal for this week. |
| A-17 | Weekly_Goal_not_achieved | You must improve to meet the weekly goal. Please stay active next week and try to overcome the shortcomings of this week. On Week-XX and Week-XY weeks, you were very active. Please try to follow similar activity patterns. |

Appendix A.3.

Table A.3: In-context recommendation conditions, and corresponding rules (rule-based) for test set-up.

| **No.** | **Semantic Rule(s) [If] and Condition [Implies]** |
| --- | --- |
| 1 | (hasActivityLevel == 0) IMPLIES (Sedentary AND hasActivityLevel)  (hasActivityLevel == 1) IMPLIES (Low_physically_active AND hasActivityLevel)  (hasActivityLevel == 2) IMPLIES (Physically_active AND hasActivityLevel)  (hasActivityLevel == 3) IMPLIES (Moderate_physically_active AND hasActivityLevel)  (hasActivityLevel == 4) IMPLIES (Vigorous_physically_active AND hasActivityLevel) |
| 2 | ((hasSedentaryBouts – daily_sedentary_goal_time as set in goal) > 0) IMLPIES (Sedentary_hour_negative)  ((hasSedentaryBouts – daily_sedentary_goal_time as set in goal) <= 0) IMLPIES (Sedentary_hour_positive) |
| 3 | ((hasSteps – daily_step_goal as set in goal) => 0) IMLPIES (Steps_positive)  ((hasSteps – daily_step_goal as set in goal) < 0) IMLPIES (Steps_negative) |
| 4 | ((hasMPAMinutes – daily_MPA_goal as set in goal) OR (hasVPAMinutes*2 – daily_VPA_goal as set in goal) => 0) IMLPIES (Activity_minute_positive)  ((hasMPAMinutes – daily_MPA_goal as set in goal) OR (hasVPAMinutes*2 – daily_VPA_goal as set in goal) < 0) IMLPIES (Activity_minute_negative) |
| 5 | ((hasWeeklyStepPrediction – weekly_step_goal as set in goal) => 0) IMLPIES (Step_forecast_trend_postive)  (hasWeeklyStepPrediction – weekly_step_goal as set in goal < 0) IMLPIES (Step_forecast_trend_negative) |
| 6 | ((hasSteps – daily_step_goal as set in goal) => 0) AND ((hasMPAMinutes – daily_MPA_goal as set in goal) OR (hasVPAMinutes*2 – daily_VPA_goal as set in goal) => 0) AND (hasTotalSleepTime => (daily_sleep_goal as set in goal *60)) AND ((hasSedentaryBouts – daily_sedentary_goal_time as set in goal) <= 0) IMLPIES (Daily_Goal_achieved) |
| 7 | ((hasSteps – weekly_step_goal as set in goal) => 0) AND ((hasMPAMinutes – weekly_MPA_goal as set in goal) OR (hasVPAMinutes*2 – weekly_VPA_goal as set in goal) => 0) AND (hasTotalSleepTime => (weekly_sleep_goal as set in goal *60)) AND ((hasSedentaryBouts – weekly_sedentary_goal_time as set in goal) <= 0) IMLPIES (Weekly_Goal_achieved) |
| 8 | (Sedentary + Low_physically_active + Moderate_physically_active + Vigorous_physically_active + Sedentary_hour_negative + Sedentary_hour_positive + Steps_negative + Steps_positive + Activity_minute_negative + Activity_minute_positive + Step_forecast_trend_postive + Step_forecast_trend_negative + Daily_Goal_achieved + Daily_Goal_not_achieved + Weekly_Goal_achieved + Weekly_Goal_not_achieved + Good_weather + Bad_weather = 1) |

Appendix A.4.

Table A.4: The attributes of MOX2-5 datasets.

| **No** | **Attributes** | **Type** | **Description** |
| --- | --- | --- | --- |
| 1 | Date | String | Recorded activity date |
| 2 | Time | String | Recorded activity time |
| 3 | UploadStatus | Character | Indicates the following two types of uploading status: ‘H’ and ‘L’. |
| 4 | IMA | Integer | Total activity intensity |
| 5 | WeightBearing | Integer | Total weight-bearing seconds |
| 6 | Sedentary | Integer | Total sedentary seconds |
| 7 | Standing | Integer | Total standing seconds |
| 8 | LPA | Integer | Total low physical activities seconds |
| 9 | MPA | Integer | Total moderate physical activities seconds |
| 10 | VPA | Integer | Total vigorous physical activities seconds |
| 11 | Steps | Integer | Total daily step count |

Appendix A.5.

Table A.5: Participant characteristics (N=16).

| **Factors** | **Mean (µ)** | **SD (σ)** | **Min** | **Max** |
| --- | --- | --- | --- | --- |
| Age | 35.375 | 7.03 | 21 | 51 |
| Height (cm) | 173.5 | 8.02 | 158.5 | 184.0 |
| Weight (Kg.) | 77.0 | 16.36 | 55.0 | 107.0 |
| BMI | 25.38 | 3.93 | 19.41 | 31.604 |
| Duration (days) | 33.6875 | 5.41 | 30 | 48 |
| Total sedentary seconds | 2449171 | 1051610.5 | 590028 | 4261190 |
| Total VPA seconds | 41887.81 | 60688.5 | 112 | 256896 |
| Total MPA seconds | 53231.75 | 17965 | 23402 | 95730 |
| Total LPA seconds | 154647.1 | 66540.6 | 32272 | 254332 |
| Total steps | 366703.3 | 87202.25 | 252551 | 588132 |

Appendix A.6.

**Figure A.7.** The training vs. testing curve for GB Classifier for original (a) PMData and (b) MOX2-5 datasets.

**
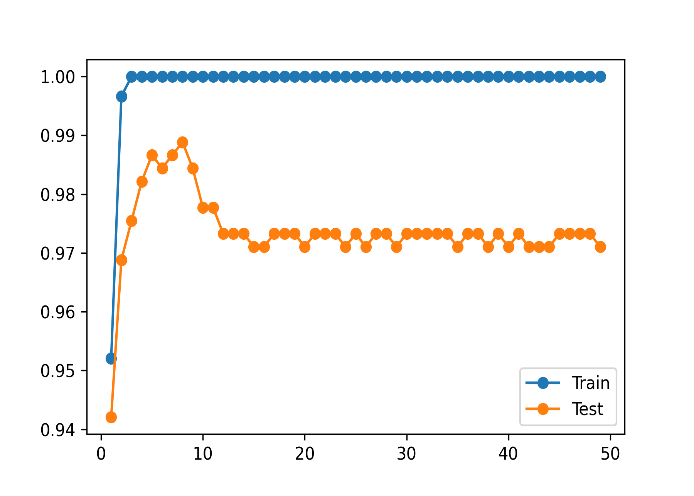

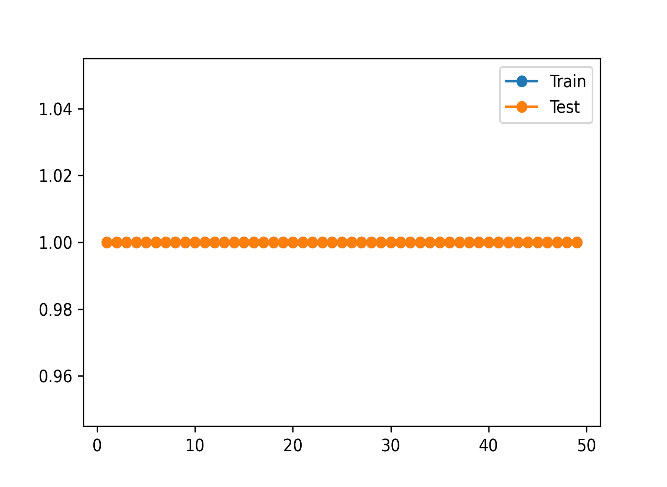
**

1. (b)

Appendix A.7.

**Figure A.7.** The learning curve for GB Classifier for original (a) PMData and (b) MOX2-5 datasets.

**
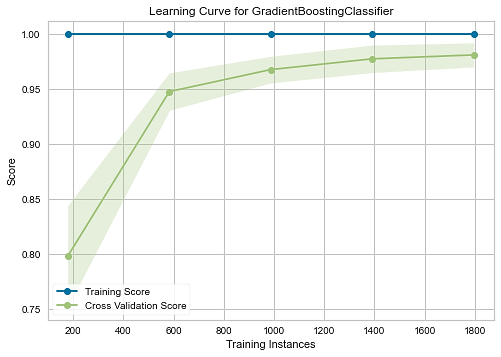

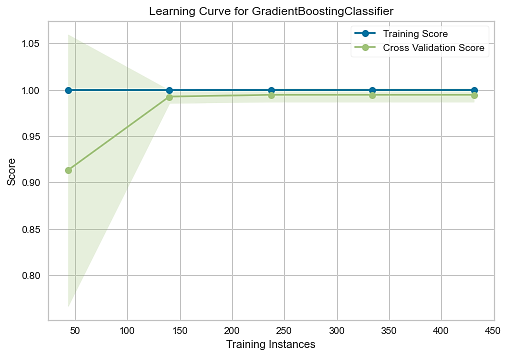
**

1. (b)

Appendix A.8.

**Figure A.8.** The validation curve for GB Classifier for original (a) PMData and (b) MOX2-5 datasets.

**
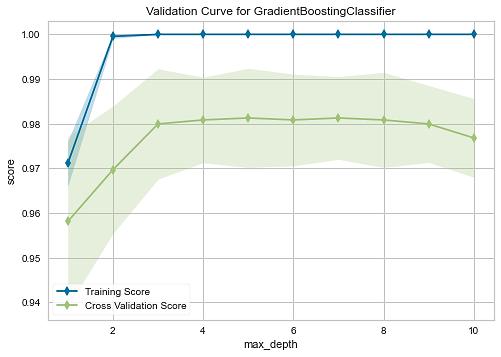

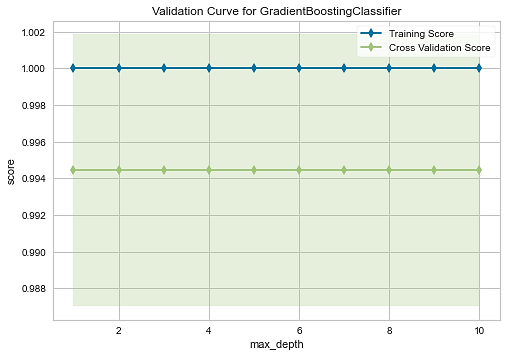
**

1. (b)

Appendix A.9.

**Figure A.9.** (a) Autocorrelation and (b) Partial autocorrelation plot for the P-1 from the original PMData dataset.


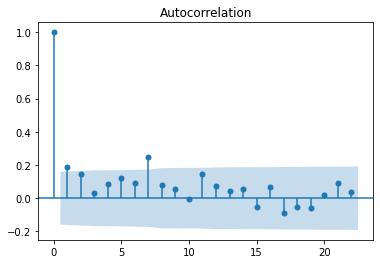

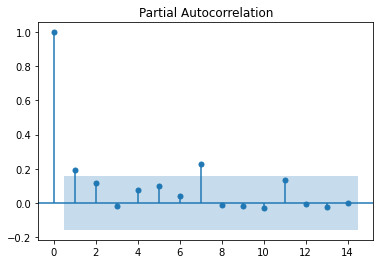


(a) (b)

Appendix A.10.

**Figure A.10.** The prediction comparison between the autoregression model with residual error minimization (predicted) and the autoregression model without residual error minimization for the P-1 from the original PMData dataset.


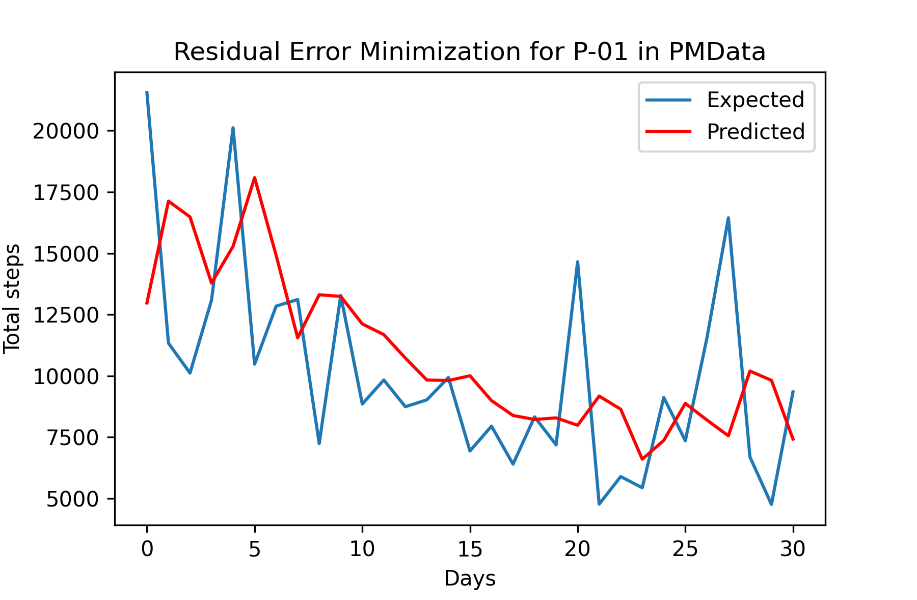


Appendix A.11.

Textbox A.11. Selected list of SPARQL queries.

| **Prefixes:**  PREFIX rdf: <http://www.w3.org/1999/02/22-rdf-syntax-ns#>  PREFIX owl: <http://www.w3.org/2002/07/owl#>  PREFIX rdfs: <http://www.w3.org/2000/01/rdf-schema#>  PREFIX xsd: <http://www.w3.org/2001/XMLSchema#>  PREFIX ssn: <http://purl.oclc.org/NET/ssnx/ssn#>  PREFIX: <http://www.co-ode.org/ontologies/uia/ont.owl#>  PREFIX status: http://www.w3.org/2003/06/sw-vocab-status/ns#  **a. Rule for viewing personal preference data:**  SELECT ?participant ?datetime ?goalTimeFrame ?goalType ?goalSteps ?goalMPA ?goalVPA ?sleepTime ?sedentaryMinutes ?physicalActivityType ?interactionFrequency ?interactionMedium ?interactionMode ?response  WHERE {  ?participant :hasPreferences ?preferences .  ?goal :hasGoalTimeFrame ?goalTimeFrame .  ?goal :hasGoalType ?goalType .  ?goal :hasGoalSteps ?goalSteps .  ?goal :hasGoalMPAMinutes ?goalMPA .  ?goal :hasGoalVPAMinutes ?goalVPA .  ?goal :totalSleepTime ?sleepTime .  ?goal :hasSedentaryMinutes ?sedentaryMinutes .  ?goal :hasPhysicalActivityType ?physicalActivityType .  ?interaction :hasInteractionFrequency ?interactionFrequency .  ?interaction :hasInteractionMedium ?interactionMedium .  ?interaction :hasInteractionMode ?interactionMode .  ?responseType :hasResponseType ?response .  ?preferences :hasTimeStamp ?temporalentity .  ?temporalentity :hasDateTime ?datetime .  }  ORDER BY DESC (?datetime)  **b. Rule for finding activity related results from an individual participant:**  SELECT ?participant ?datetime ?status ?level ?day1 ?day2 ?day3 ?day4 ?day5 ?day6 ?day7  WHERE {  ?participant :hasStatus ?participantStatus .  ?participantStatus :hasParticipantStatus ?status .  ?participant :hasHealthRecord ?participanthealthrecord .  ?activityLevel :hasActivityLevel ?level .  ?stepPrediction : hasIntervalDay1 ?day1 .  ?stepPrediction : hasIntervalDay2 ?day2 .  ?stepPrediction : hasIntervalDay3 ?day3 .  ?stepPrediction : hasIntervalDay4 ?day4 .  ?stepPrediction : hasIntervalDay5 ?day5 .  ?stepPrediction : hasIntervalDay6 ?day6 .  ?stepPrediction : hasIntervalDay7 ?day7 .  ?participanthealthrecord :hasTimeStamp ?temporalentity .  ?temporalentity :hasDateTime ?datetime .  }  ORDER BY DESC (?datetime)  **c. Rule for finding recommendation results from an individual participant:**  SELECT ?participant ?datetime ?action ?element ?subject ?time ?formalMessages ?informalMessages  WHERE {  ?participant :hasReceivedRecommendation ?recommendation .  ?messageComponent :hasAction ?action .  ?messageComponent :hasElement ?element .  ?messageComponent :hasSubject ?subject .  ?messageComponent :hasTime ?time .  ?formal :hasRecommendationMessages ?formalMessages .  ?informal :hasRecommendationMessages ?informalMessages .  ?recommendation :hasTimeStamp ?temporalentity .  ?temporalentity :hasDateTime ?datetime .  }  ORDER BY DESC (?datetime) |
| --- |
